# Recessive, pathogenic *AARS1* variants display variable loss-of-function and dominant-negative effects

**DOI:** 10.1242/dmm.052006

**Published:** 2025-06-27

**Authors:** Molly E. Kuo, Kira E. Jonatzke, Maclaine Parish, Anthony Antonellis

**Affiliations:** ^1^ Department of Human Genetics, University of Michigan, Ann Arbor, MI 48109, USA; ^2^ Medical Scientist Training Program, University of Michigan, Ann Arbor, MI 48109, USA; ^3^ Cellular and Molecular Biology Program, University of Michigan, Ann Arbor, MI 48109, USA; ^4^ Department of Neurology, University of Michigan, Ann Arbor, MI 48109, USA

**Keywords:** Aminoacyl-tRNA synthetases, Mendelian disease, Humanized yeast models

## Abstract

Alanyl-tRNA synthetase 1 (*AARS1*) has been implicated in multi-system recessive phenotypes and in later-onset dominant neuropathy; to date, no single variant has been associated with both dominant and recessive diseases, raising questions about shared mechanisms between the two inheritance patterns. *AARS1* variants associated with recessive disease result in loss-of-function or hypomorphic alleles, and this has been demonstrated, in part, via yeast complementation assays. However, pathogenic alleles have not been assessed in a side-by-side study. Here, we employed a humanized yeast model to evaluate the functional consequences of all *AARS1* missense variants reported in recessive disease. The majority of variants showed variable loss-of-function effects, ranging from no growth to significantly reduced growth. These data deem yeast a reliable model to test the effects of *AARS1* variants; however, our data also indicate that this model is prone to false-negative results and is not informative for genotype–phenotype studies. We next tested missense variants associated with no growth for dominant-negative effects. Interestingly, K81T and E99G *AARS1* demonstrated both loss-of-function and dominant-negative effects, indicating that certain *AARS1* variants can cause both dominant and recessive disease phenotypes.

## Teaser

**Summary:** This study uses a humanized yeast model to test the functional consequences of recessive, pathogenic *AARS1* alleles. The data reveal that certain alleles have dominant-negative effects, indicating that carriers of these recessive variants are susceptible to dominant *AARS1*-associated neuropathy.

## INTRODUCTION

Aminoacyl-tRNA synthetases (ARSs) are essential, ubiquitously expressed enzymes that charge transfer RNA (tRNA) molecules with cognate amino acids (Antonellis and Green, 2008). There are 37 ARS loci in the human nuclear genome, which encode enzymes that function in the cytoplasm or mitochondria (Antonellis and Green, 2008). Variants in ARS genes have been implicated in (1) recessive diseases with varying clinical presentations that often include early-onset, multi-system, neurodevelopmental phenotypes; and (2) dominant axonal peripheral neuropathies, also called Charcot-Marie-Tooth disease (Meyer-Schuman and Antonellis, 2017). Previous genetic and functional data showed that a partial loss-of-function effect is the molecular mechanism of ARS-mediated recessive disease (Meyer-Schuman and Antonellis, 2017). Indeed, patients often carry one nonfunctional allele and one hypomorphic allele; complete ablation of the function of any ARS would be lethal. Variants implicated in ARS-mediated dominant peripheral neuropathy affect homodimeric ARS enzymes, demonstrate loss-of-function and/or gain-of-function effects without significantly decreasing protein expression (Griffin et al., 2014; He et al., 2015), and activate the integrated stress response (Spaulding et al., 2021). We and others have presented data indicating a dominant-negative mechanism for neuropathy-associated ARS variants (Mullen et al., 2020; Meyer-Schuman et al., 2023); however, the manner in which this mechanism intersects with the integrated stress response remains unclear.

Alanyl-tRNA synthetase 1 [*AARS1*; Online Mendelian Inheritance in Man (OMIM) 601065] encodes the cytoplasmic enzyme that charges tRNA^Ala^ with alanine (Antonellis and Green, 2008). Variants in *AARS1* have been associated with multi-system recessive phenotypes and with dominant axonal peripheral neuropathy; however, no single variant has been reported to cause both dominant and recessive disease (Meyer-Schuman and Antonellis, 2017). Bi-allelic *AARS1* variants have been identified in a spectrum of recessive disease phenotypes including (1) epileptic encephalopathy, hypomyelination and progressive microcephaly; (2) tetraparesis; (3) recurrent acute liver failure; and (4) non-photosensitive trichothiodystrophy (Table 1) (Simons et al., 2015; Nakayama et al., 2017; Marten et al., 2020; Botta et al., 2021; Helman et al., 2021). The functional consequences of a subset of variants have been studied in RNA expression studies, western blot analyses, *in vitro* aminoacylation assays and/or yeast complementation assays (Table 2) (Simons et al., 2015; Nakayama et al., 2017; Marten et al., 2020; Botta et al., 2021; Helman et al., 2021). However, pathogenic *AARS1* variants have not been compared in a side-by-side manner to assess the effectivity of each assay to detect loss-of-function effects. Addressing this issue is important for identifying a pipeline of informative functional assays that will aid in building or refuting arguments for the pathogenicity of newly identified variants.

**Table 1.** AARS1 variants identified in patients with recessive disease phenotypes

Family	Number of patients	Nucleotide changes*	Amino acid changes^‡^	Phenotype	Reference
A	2	c.242A>C/c.2251A>G	p.Lys81Thr/p.Arg751Gly	Epileptic encephalopathy, myelination defect	Simons et al., 2015
B	1	c.2251A>G/c.2251A>G	p.Arg751Gly/p.Arg751Gly	Epileptic encephalopathy, myelination defect	Simons et al., 2015
C	2	c.2067dupC/c.2738G>A	p.Tyr690Leufs*3/p.Gly913Asp	Microcephaly, hypomyelination, epileptic encephalopathy	Nakayama et al., 2017
D	1	c.893T>A/c.2251A>G	p.Leu298Gln/p.Arg751Gly	Recurrent acute liver failure	Marten et al., 2020
E	1	c.2096T>C/c.2702G>A	p.Ile699Thr/p.Cys901Tyr	Non-photosensitive trichothiodystrophy	Botta et al., 2021
F	1	c.2176A>G/c.2267C>T	p.Thr726Ala/p.Thr756Ile	Non-photosensitive trichothiodystrophy	Botta et al., 2021
G	1	c.296A>G/c.778A>G	p.Glu99Gly/p.Thr260Ala	Late-onset progressive cognitive decline, gait imbalance, peripheral vision loss, dysarthria	Helman et al., 2021
H	1	c.562_563delinsCA/c.1574G>A	p.Ser188His/p.Cys525Tyr	Intrauterine growth restriction, spastic tetraparesis, optic atrophy, focal epilepsy	Helman et al., 2021
I	1	c.1741G>A/c.1741G>A	p.Gly581Ser/p.Gly581Ser	Spastic tetraparesis, focal epilepsy, microcephaly	Helman et al., 2021
J	1	c.1997T>C/exon 1-4 deletion	p.Val666Ala/p.?	Late onset tetraparesis, bradykinesia, dystonic movements	Helman et al., 2021
K	1	c.2251A>G/c.1812C>G	p.Arg751Gly/p.Asn604Lys	Epileptic encephalopathy, developmental delay, microcephaly, hypotonia	Helman et al., 2021
L	2	c.2286G>A/c.2286G>A	p.(Lys762=Ala763Valfs*11)/p.(Lys762=Ala763Valfs*11)	Epileptic encephalopathy, developmental delay, microcephaly, hypotonia	Helman et al., 2021
M	1	c.988C>T/c.2738G>A	p.Arg330*/p.Gly913Asp	Epileptic encephalopathy, developmental delay, microcephaly, hypotonia	Helman et al., 2021
N	1	c.410_413del/c.1589A>G	p.Tyr137Leufs*9/p.Asp530Gly	Epileptic encephalopathy, developmental delay, microcephaly, hypotonia	Helman et al., 2021
O	1	c.462_463del/c.1741G>A	p.Gln154Hisfs*9/p.Gly581Ser	Epileptic encephalopathy, developmental delay, microcephaly, hypotonia	Helman et al., 2021
P	1	c.997C>T/c.2738G>A	p.Arg333*/p.Gly913Asp	Epileptic encephalopathy, developmental delay, microcephaly, hypotonia	Helman et al., 2021

*Human *AARS1* nucleotide positions correspond to GenBank accession number NM_001605.3. ^‡^Human AARS1 amino acid positions correspond to GenBank accession number NP_001596.2. ^§^Functional data inferred from G757* (Meyer-Schuman et al., 2023).

**Table 2.** Functional consequences of disease-associated AARS1 variants

Family	Amino acid change	Detection in gnomAD*	AlphaMissense prediction^‡^	*In vitro* tRNA charging	Yeast complementation: modeled in *AARS1*/pAG425	Yeast complementation: modeled in *AARS1*/p413	Growth curve: modeled in *AARS1*/pAG425
A	p.Lys81Thr	1/152,164	.994 – likely pathogenic	2-fold decrease (Simons et al., 2015)	LOF	LOF	Not tested
	p.Arg751Gly	218/1,614,142	.995 – likely pathogenic	10-fold decrease (Simons et al., 2015)	Normal	Normal	Normal
B	p.Arg751Gly	218/1,614,142	.995 – likely pathogenic	10-fold decrease (Simons et al., 2015)	Normal	Normal	Normal
	p.Arg751Gly	218/1,614,142	.995 – likely pathogenic	10-fold decrease (Simons et al., 2015)	Normal	Normal	Normal
C	p.Tyr690Leufs*3	None reported	Not applicable	86% decreased catalytic efficiency (*k*_cat_/*K*_M_) (Nakayama et al., 2017)	Not tested (null)	Not tested (null)	Not tested
	p.Gly913Asp	28/1,614,066	.787 – likely pathogenic	73% decreased catalytic efficiency (*k*_cat_/*K*_M_) (Nakayama et al., 2017)	Normal	Hypomorph	Normal
D	p.Leu298Gln	1/628,774	.978 – likely pathogenic	Not tested	Normal	Normal	Hypomorph
	p.Arg751Gly	218/1,614,142	.995 – likely pathogenic	10-fold decrease (Simons et al., 2015)	Normal	Normal	Normal
E	p.Ile699Thr	5/1,461,670	.567 – likely pathogenic	Not tested	Normal	Hypomorph	Normal
	p.Cys901Tyr	None reported	.956 – likely pathogenic	Not tested	LOF	Not tested	Normal
F	p.Thr726Ala	6/1,613,946	.694 – likely pathogenic	Not tested	Hypomorph	Not tested	Not tested
	p.Thr756Ile	40/1,614,030	.979 – likely pathogenic	Not tested	Normal	Normal	Normal
G	p.Glu99Gly	None reported	.988 – likely pathogenic	Not tested	LOF	Not tested	Not tested
	p.Thr260Ala	2/628,782	.699 – likely pathogenic	Not tested	Normal	Normal	Normal
H	p.Ser188His	None reported	Not applicable	Not tested	Hypomorph	Not tested	Not tested
	p.Cys525Tyr	None reported	.858 – likely pathogenic	Not tested	Normal	Normal	Normal
I	p.Gly581Ser	3/1,461,618	.476 – ambiguous	Not tested	Normal	Hypomorph	Normal
	p.Gly581Ser	3/1,461,618	.476 – ambiguous	Not tested	Normal	Hypomorph	Normal
J	p.Val666Ala	4/1,589,084	.846 – likely pathogenic	Not tested	Hypomorph	Not tested	Not tested
	Exon 1-4 deletion; p.?	Not available	Not applicable	Not tested	Not tested (null)	Not tested (null)	Not tested
K	p.Arg751Gly	218/1,614,142	.995 – likely pathogenic	10-fold decrease (Simons et al., 2015)	Normal	Normal	Normal
	p.Asn604Lys	None reported	.976 – likely pathogenic	Not tested	Normal	LOF	Not tested
L	p.(Lys762=Ala763Valfs*11)	1/628,716	Not applicable	Not tested	Not tested	Not tested	Not tested
	p.(Lys762=Ala763Valfs*11)	1/628,716	Not applicable	Not tested	Not tested	Not tested	Not tested
M	p.Arg330*	7/1,614,014	Not applicable	Not tested	Not tested (null)	Not tested (null)	Not tested
	p.Gly913Asp	28/1,614,066	.787 – likely pathogenic	Not tested	Normal	Hypomorph	Normal
N	p.Tyr137Leufs*9	None reported	Not applicable	Not tested	Not tested (null)	Not tested (null)	Not tested
	p.Asp530Gly	2/780,962	.42 – ambiguous	Not tested	Normal	Normal	Normal
O	p.Gln154Hisfs*9	None reported	Not applicable	Not tested	Not tested (null)	Not tested (null)	Not tested
	p.Gly581Ser	3/1,461,618	.476 – ambiguous	Not tested	Normal	Hypomorph	Normal
P	p.Arg333*	5/1,461,850	Not applicable	Not tested	Not tested (null)	Not tested (null)	Not tested
	p.Gly913Asp	28/1,614,066	.787 – likely pathogenic	Not tested	Normal	Hypomorph	Normal

LOF, loss of function; tRNA, transfer RNA. *Allele frequencies were determined using gnomAD v4.0.0. ^‡^AlphaMissense (accessed on 1 March 2024) (Cheng et al., 2023).

In this study, we employed a humanized yeast model to evaluate the functional consequences of 16 *AARS1* missense variants associated with recessive disease, assuming that all identified early frameshift, nonsense and large-deletion mutations represent null alleles. Each missense variant was individually tested in one or two yeast complementation assays, which employed high- or low-copy number expression vectors. Subsequently, three nonfunctional missense variants were evaluated for dominant-negative effects by testing for effects on protein expression and the ability of each to repress a wild-type copy of human *AARS1*. Our data revealed that yeast is an informative model system to test human *AARS1* variants for loss-of-function effects in the context of recessive disease. These data also revealed important limitations of yeast as a model system, including inability to detect subtle deficits in gene function and to explain genotype–phenotype relationships. It will be important to consider these limitations in future uses of yeast to study pathogenic *AARS1* alleles. In addition, our data suggest that all loss-of-function *AARS1* missense variants have the potential to exert dominant-negative effects; but, for some variants, this is ameliorated through reduced protein expression or reduced dimerization with the wild-type subunit. These findings suggest that some carriers of pathogenic *AARS1* missense alleles that cause recessive disease (i.e. the parents and siblings of affected individuals) manifest *AARS1*-associated dominant axonal neuropathy. In sum, our findings have important implications for studying the allelic and clinical heterogeneity – as well as the mechanisms – of *AARS1*-associated inherited disease.

## RESULTS

### A collection of *AARS1* variants currently implicated in recessive disease

**Fig. 1.**
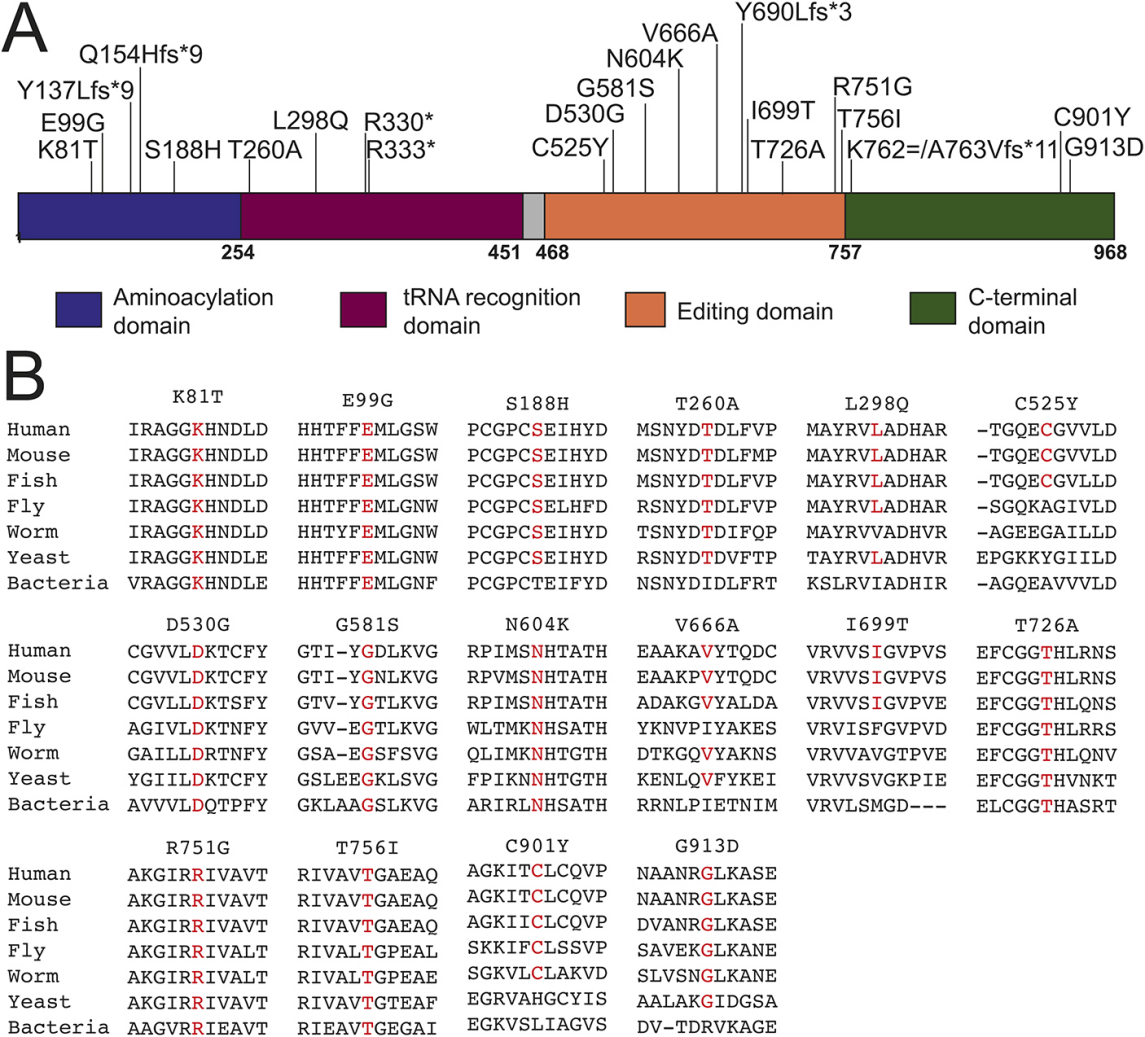
**Localization and conservation of *AARS1* variants implicated in recessive disease**. (A) AARS1 functional domains are indicated in blue (aminoacylation domain), purple [transfer RNA (tRNA) recognition domain], orange (editing domain) and green (C-terminal domain). The positions of the variants are shown across the top, and numbers along the bottom indicate amino acid positions. The exon 1-4 deletion from Family J is not depicted. (B) Conservation of the affected amino acid residues for all missense variants implicated in recessive disease. The position of each variant is shown along with flanking AARS1 amino acid residues from multiple, evolutionarily diverse species. The wild-type human amino acid residue at the position of the affected residue is shown in red for each species.

To collect all reported recessive disease-associated *AARS1* alleles, we performed a literature review, which revealed 23 variants (Table 1) (Simons et al., 2015; Nakayama et al., 2017; Marten et al., 2020; Botta et al., 2021; Helman et al., 2021). Seven variants result in premature stop codons or large deletions, and we assume that these genetic lesions result in null alleles. This assumption is based, in part, on our previous observation that the G757* engineered *AARS1* allele results in no detectable protein via western blot analysis (Meyer-Schuman et al., 2023) and that the 3′-most premature stop codon occurs at A763. In addition, 16 of the reported disease-associated variants are missense changes. The AARS1 protein contains (1) an aminoacylation domain for activating and transferring the amino acid to tRNA; (2) a tRNA recognition domain; (3) an editing domain for hydrolyzing mischarged tRNA; and (4) a C-terminal domain that is important for dimerization (Naganuma et al., 2009). The variants map throughout the functional domains. Of the missense variants, three are located in the aminoacylation domain, two are located in the tRNA recognition domain, nine are located in the editing domain, and two are located in the C-terminal domain (Fig. 1A). The affected residues for each of the 16 missense variants show variable conservation, with all residues conserved among human, mouse and fish (Fig. 1B). Each variant was either not present or present at a low frequency (<0.0002) in gnomAD, and no homozygous individuals were noted (Table 2). Combined, these data are consistent with the disease-associated *AARS1* missense variants being pathogenic and causing loss-of-function effects.

### *AARS1* variants implicated in recessive disease show loss-of-function effects in yeast complementation assays

**Fig. 2.**
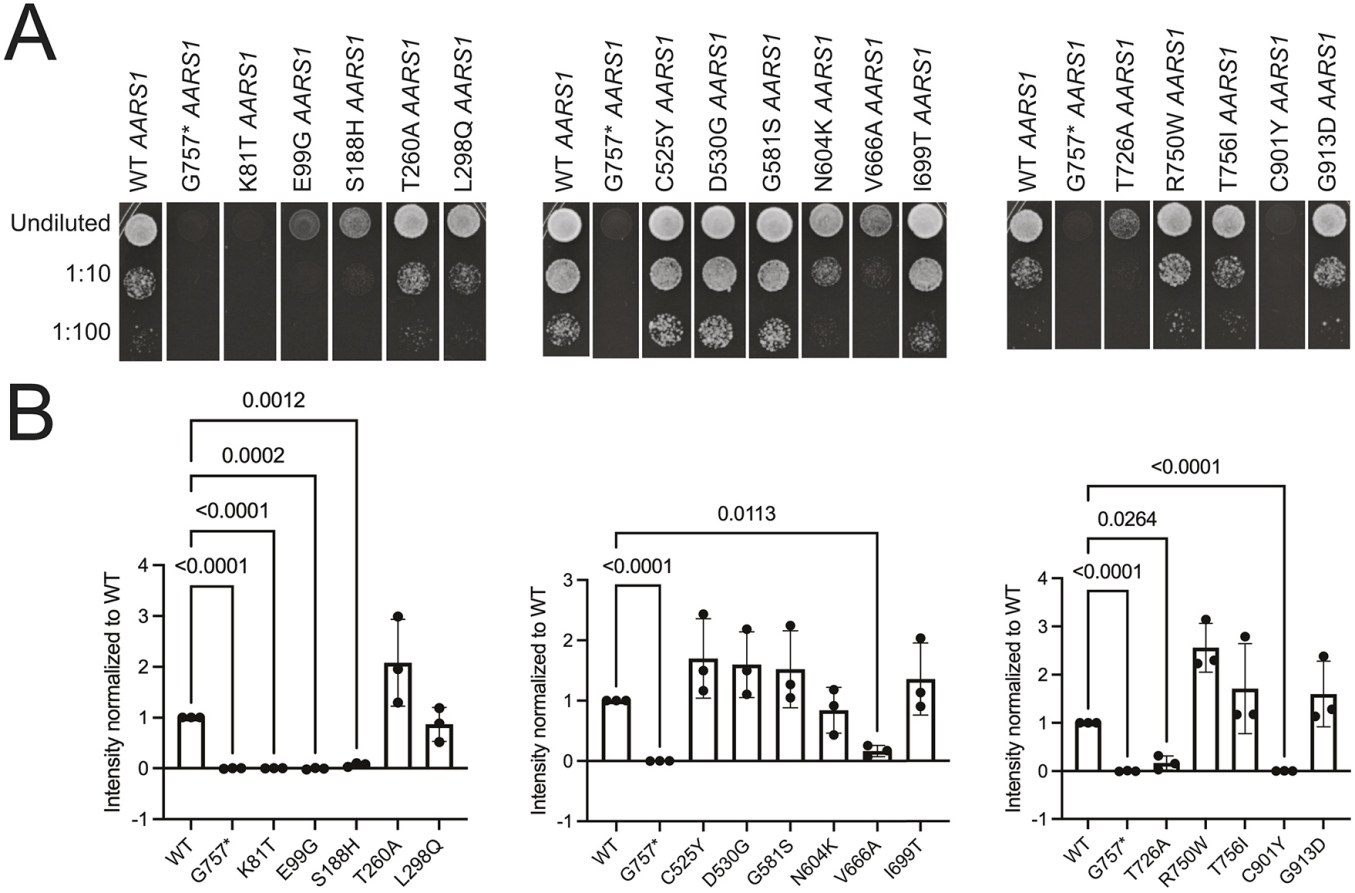
**Assessment of loss-of-function effects of *AARS1* missense variants in yeast complementation assays using a high-copy number vector (pAG425).** Haploid yeast with a doxycycline-repressible endogenous *ALA1* (the yeast ortholog of *AARS1*) were transformed with pAG425 vectors containing wild-type (WT) or mutant *AARS1*, or a vector with a null allele (G757* *AARS1*); the variant studied in each experiment is indicated. (A) Resulting cultures were plated undiluted or diluted (1:10 or 1:100) on media containing doxycycline and grown at 30°C for 5 days. (B) Images of yeast spots were analyzed to assess the relative growth of mutant variants in comparison to WT *AARS1.* The average growth rate across three colony replicates for each mutant was calculated and is depicted as the bar height. Error bars represent s.d. Statistical significance was determined by one-way ANOVA with the Geisser-Greenhouse correction and Dunnett's multiple comparison's test with individual variances computed for each comparison. Only comparisons that were statistically significant are annotated with a *P*-value.

To test and compare the functional consequences of the 16 disease-associated *AARS1* missense variants, we employed a humanized yeast complementation assay and tested the ability of each variant to complement loss of the endogenous yeast gene, *ALA1*. Briefly, we used a haploid yeast strain (ptetO7-*ALA1*) with endogenous yeast *ALA1* under the control of a tetracycline-repressible promoter (Meyer-Schuman et al., 2023). Wild-type *AARS1*, mutant *AARS1* or a previously reported null allele (G757*) were cloned into the pAG425 vector, a high-copy number vector with a galactose-inducible promoter. These expression constructs were transformed into the yeast strain, and yeast growth was evaluated on medium containing doxycycline (to represses endogenous *ALA1* expression) and galactose (to expresses *AARS1* from pAG425). Null *AARS1* did not support yeast growth (Fig. 2A,B; Fig. S3), consistent with *ALA1* being an essential gene. Wild-type human *AARS1* expression supported yeast growth (Fig. 2A,B; Fig. S3), indicating that human *AARS1* can complement the loss of the endogenous *ALA1* locus; however, this rescue was reduced compared to rescue with yeast *ALA1* on the same plasmid (Fig. S1), which is consistent with previous findings (Meyer-Schuman et al., 2023). Three human missense variants (K81T, E99G and C901Y *AARS1*) were unable to support yeast growth (Fig. 2A,B; Fig. S3), indicating that these variants represent complete loss-of-function alleles. Three additional human variants (S188H, V666A and T726A *AARS1*) supported growth that was significantly reduced compared to that by wild-type *AARS1* (*P*<0.05; Fig. 2A,B; Fig. S3), indicating that these variants are hypomorphic alleles. The ten remaining human variants (T260A, L298Q, C525Y, D530G, G581S, N604K, I699T, R751G, T756I and G913D *AARS1*) supported growth in a manner similar to wild-type *AARS1* (Fig. 2A,B; Fig. S3).

**Fig. 3.**
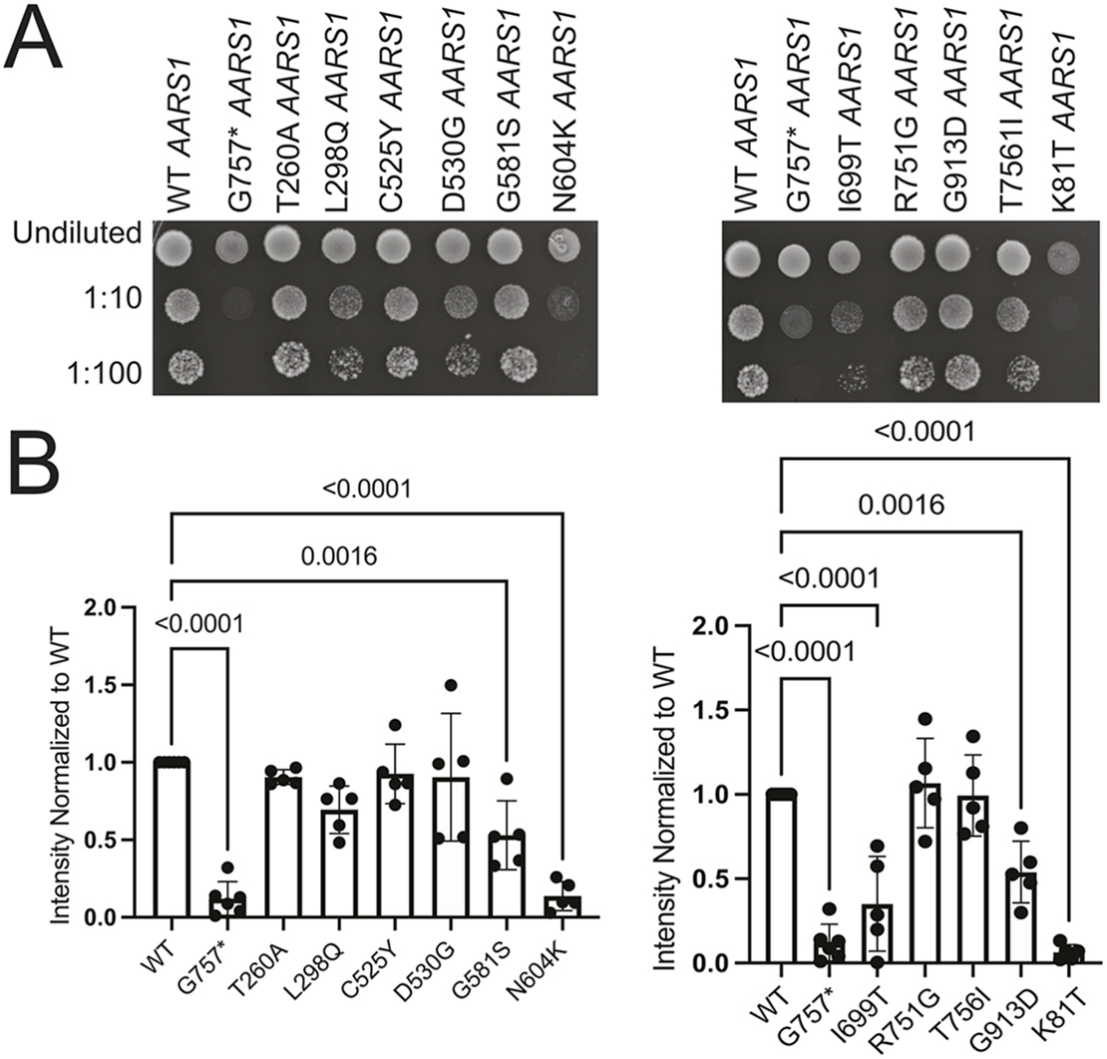
**Assessment of loss-of-function effects of *AARS1* missense variants in yeast complementation assays using a low-copy number vector (p413).** Haploid yeast with a doxycycline-repressible endogenous *ALA1* (the yeast ortholog of *AARS1*) were transformed with p413 vectors containing WT or mutant *AARS1*, or a null allele (G757* *AARS1*); the variant studied in each experiment is indicated. (A) Resulting cultures were plated undiluted or diluted (1:10 or 1:100) on media containing doxycycline and grown at 30°C for 5 days. (B) Images of yeast spots were analyzed to assess the relative growth of mutant variants in comparison to WT *AARS1.* The average growth rate across three colony replicates for each mutant was calculated and is depicted as the bar height. Error bars represent s.d. Statistical significance was determined by one-way ANOVA with the Geisser-Greenhouse correction and Dunnett's multiple comparison's test with individual variances computed for each comparison. Only comparisons that were statistically significant are annotated with a *P*-value.

In the above experiments, each variant was expressed from a high-copy number plasmid, raising the possibility of false-negative results (i.e. by masking subtle loss-of-function effects). For the ten missense variants that supported similar growth to wild-type *AARS1* in the above system, we evaluated effects on yeast growth using a low-copy number expression vector. Here, wild-type, mutant and null (G757*) human *AARS1* were cloned into p413, a low-copy number plasmid. The p413 expression constructs were then transformed into the ptetO7-*ALA1* haploid yeast strain, and growth was evaluated on doxycycline-containing medium to repress endogenous *ALA1*. Four human alleles (G581S, N604K, I699T and G913D *AARS1*) supported growth that was significantly reduced compared to that by wild-type *AARS1* (Fig. 3A,B; Fig. S4); combined with the results from the high-copy expression vector, these data indicate that these four variants are hypomorphic alleles. The six remaining human alleles (T260A, L298Q, C525Y, D530G, R751G and T756I *AARS1*) supported growth similarly to wild-type human *AARS1* (Fig. 3A,B; Fig. S4).

**Fig. 4.**
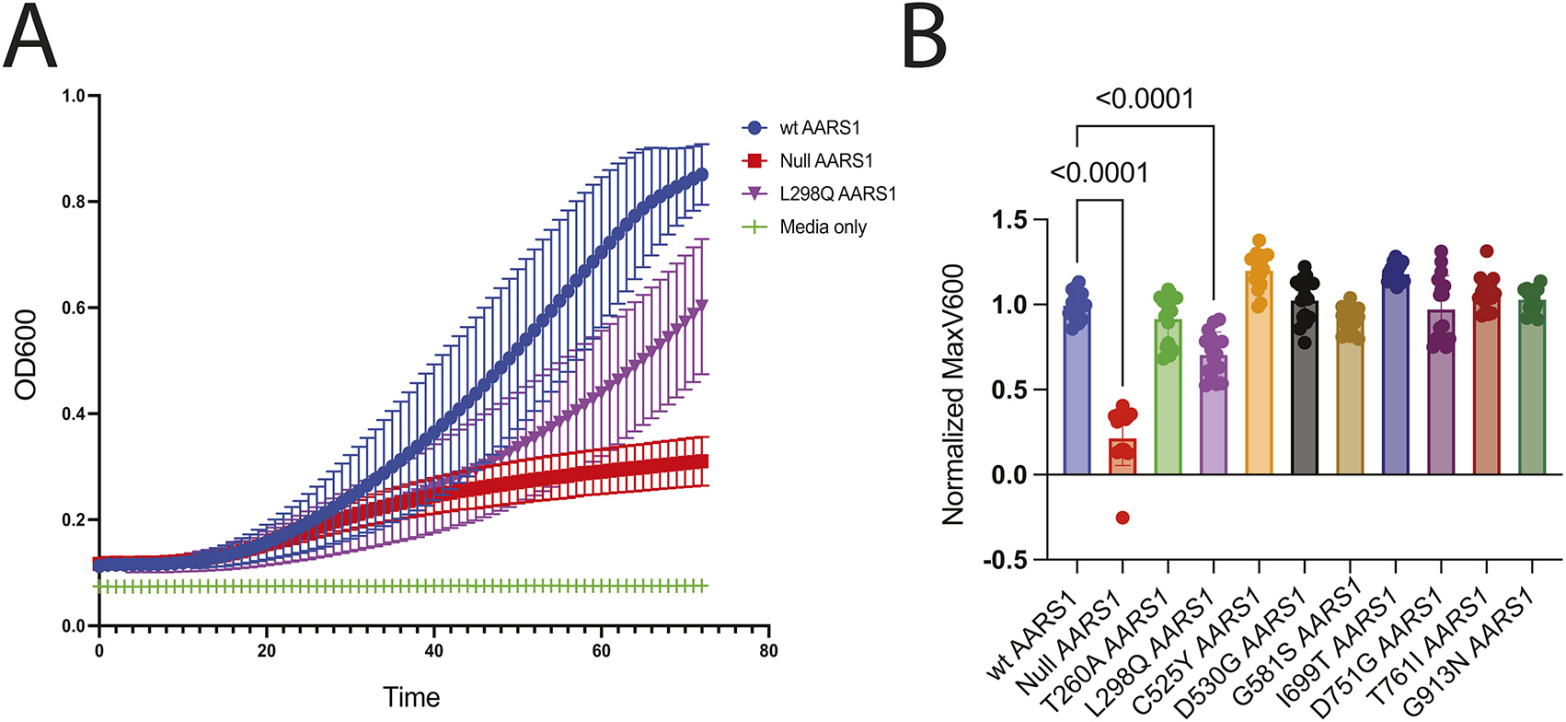
**Growth curve assessments of loss-of-function effects of *AARS1* missense variants in yeast growth assays using a high-copy number, galactose-inducible vector (pAG425).** (A) Haploid yeast with a doxycycline-repressible endogenous *ALA1* (the yeast ortholog of *AARS1*) were transformed with pAG425 vectors containing WT *AARS1* or mutant *AARS1*, or a vector with a null allele (G757* *AARS1*). Resulting cultures were diluted with media containing 2% galactose/1% raffinose, lacking leucine, and containing 100 μg/ml doxycycline to a starting optical density at 600 nm (OD600) of 0.1. Cultures were grown for 72 h with readings taken every hour. Error bars represent s.d. (B) The maximum OD600 (MaxV600) was recorded from the growth curves, and the background was subtracted and then normalized to the average WT value on the plate, which was set to one. Error bars represent s.d. Statistical significance was determined by one-way ANOVA with the Geisser-Greenhouse correction and Dunnett's multiple comparison's test with individual variances computed for each comparison. Only comparisons that were statistically significant are annotated with a *P*-value.

To quantify growth associated with pathogenic *AARS1* alleles, nine variants that showed no loss-of-function effects compared to wild-type *AARS1* or that were hypomorphic in the low-copy number system (T260A, L298Q, C525Y, D530G, G581S, I699T, R571G, T756I and G913D) were expressed from a high-copy number, galactose-inducible vector at 30°C for 72 h in selective media with optical density at 600 nm (OD600) readings taken every hour. This experiment revealed one additional hypomorphic allele (L298Q; Fig. 4; Fig. S6). Thus, the humanized yeast complementation assay described above was able to detect variable loss-of-function effects for 11 out of 16 (68.75%) disease-associated *AARS1* missense variants.

### Expression of protein from K81T and E99G *AARS1* is comparable to that from wild-type *AARS1* in yeast assays

Loss-of-function effects have been described for *AARS1* alleles associated with recessive syndromes and with dominant axonal neuropathy (McLaughlin et al., 2012; Simons et al., 2015). Although a single *AARS1* allele has not been implicated in both recessive and dominant phenotypes, the neuropathy is later onset and may not have manifested at time of examination in the parents or siblings of individuals affected with the *AARS1*-related recessive disease. Additionally, a later-onset peripheral neuropathy might not be a primary concern for families with members affected with *AARS1*-related recessive phenotypes. Previously, we demonstrated that neuropathy-associated *AARS1* alleles have dominant-negative properties in a humanized yeast assay (Meyer-Schuman et al., 2023). To determine whether certain recessive *AARS1* alleles also have dominant-negative properties, we studied the three missense *AARS1* variants that were unable to support any yeast cell growth: K81T, E99G and C901Y.

**Fig. 5.**
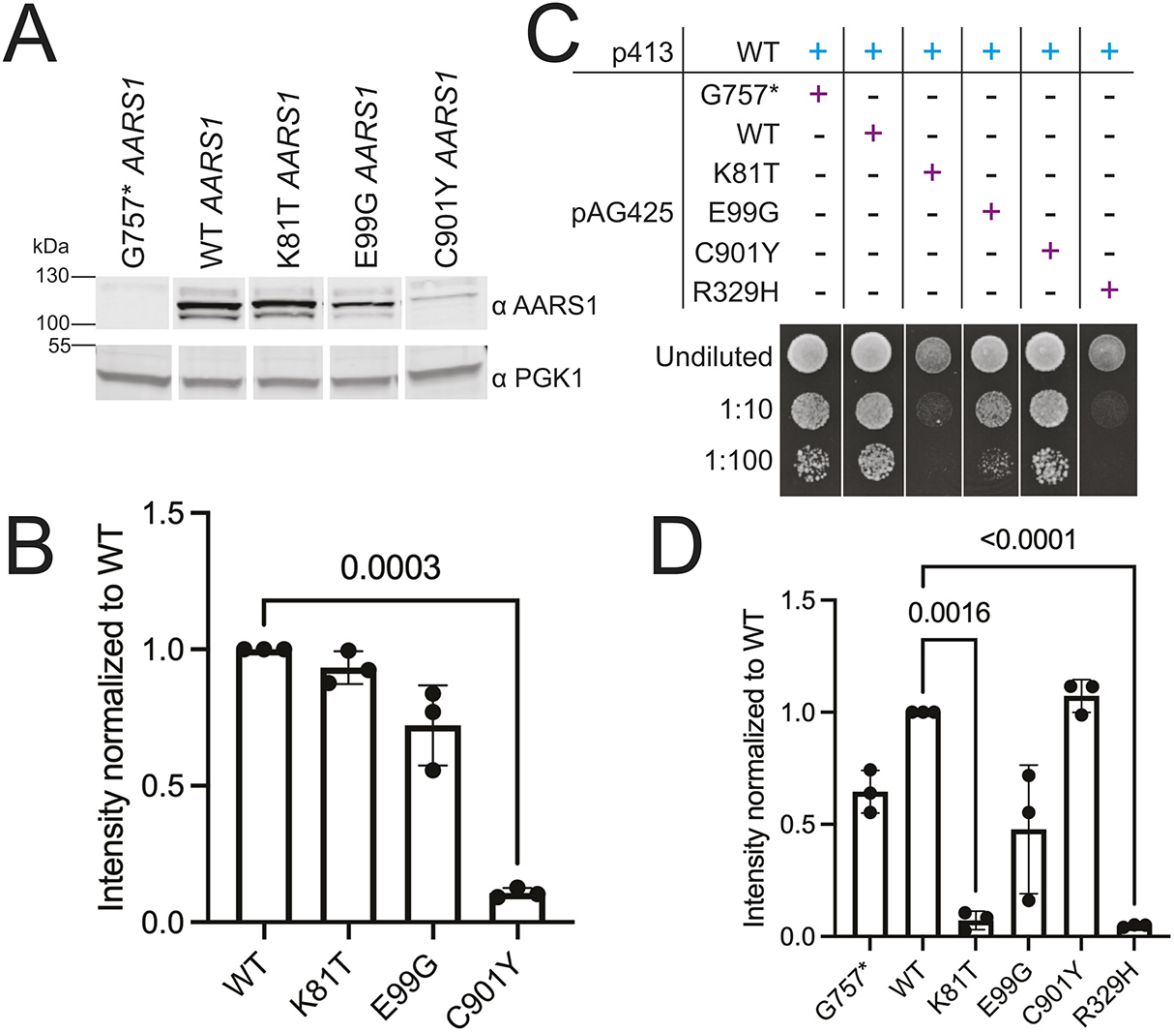
**Protein expression and dominant-negative effects of loss-of-function *AARS1* missense variants**. (A) AARS1 protein expression in transformed yeast. Western blot analyses were performed on protein lysates isolated from haploid yeast that were transformed with vectors containing the indicated inserts. The vector used in each experiment is across the top, and sizes (kDa) are indicated on the left. (B) Band intensity was quantified relative to control PGK1 levels and normalized to WT AARS1 protein levels. (C) Haploid yeast with a doxycycline-repressible endogenous *ALA1* (the yeast ortholog of *AARS1*) and containing a WT *AARS1* p413 construct were transformed with pAG425 vectors containing the indicated *AARS1* allele. Resulting cultures were plated undiluted or diluted (1:10 or 1:100) on media containing doxycycline and grown at 30°C for 5 days. (D) Images of yeast spots were analyzed to assess the relative growth of yeast co-expressing WT and *AARS1* mutants in comparison to yeast co-expressing WT *AARS1* on both p413 and pAG425. In B and D, the average intensity for three replicates was calculated and is depicted as the bar height. Error bars represent s.d. Statistical significance was determined by one-way ANOVA with the Geisser-Greenhouse correction and Dunnett's multiple comparison's test with individual variances computed for each comparison. Only comparisons that were statistically significant are annotated with a *P*-value.

For an allele to have dominant-negative properties, the gene product should be expressed and able to interact with the wild-type protein, but the gene product should be nonfunctional (Veitia, 2007). To assess this for the three human *AARS1* alleles under study, we performed western blot analyses on protein isolated from yeast transformed with pAG425 vectors to express wild-type, null or mutant human *AARS1*. We used antibodies against AARS1 and phosphoglycerate kinase (PGK1), which was utilized as a yeast cell loading control. In protein isolated from yeast transformed with G757* *AARS1*, there was no band, consistent with G757* *AARS1* being a null allele and resulting in no detectable protein (Fig. 5A,B; Fig. S7). In protein isolated from yeast transformed with wild-type *AARS1*, there was a band between 100 and 130 kDa, which is consistent with the predicted molecular mass of the AARS1 protein, ∼107 kDa (Fig. 5A,B; Fig. S7). In yeast transformed with K81T or E99G *AARS1*, AARS1 protein band intensity was similar to that in yeast transformed with wild-type *AARS1*, suggesting that these two variants do not disrupt AARS1 protein expression (Fig. 5A,B; Fig. S7; note that the mean E99G protein level was decreased but not significantly different from the wild-type protein level). Finally, in protein isolated from yeast transformed with C901Y *AARS1*, AARS1 protein band intensity was significantly reduced compared to that in yeast transformed with wild-type *AARS1* (*P*=0.0003; Fig. 5A,B; Fig. S7). In summary, these studies show that K81T and E99G AARS1 are loss-of-function proteins that are expressed at levels comparable to wild-type protein levels and could therefore interact with wild-type AARS1.

### K81T and E99G *AARS1* repress the function of wild-type *AARS1* in yeast growth assays

**Fig. 6.**
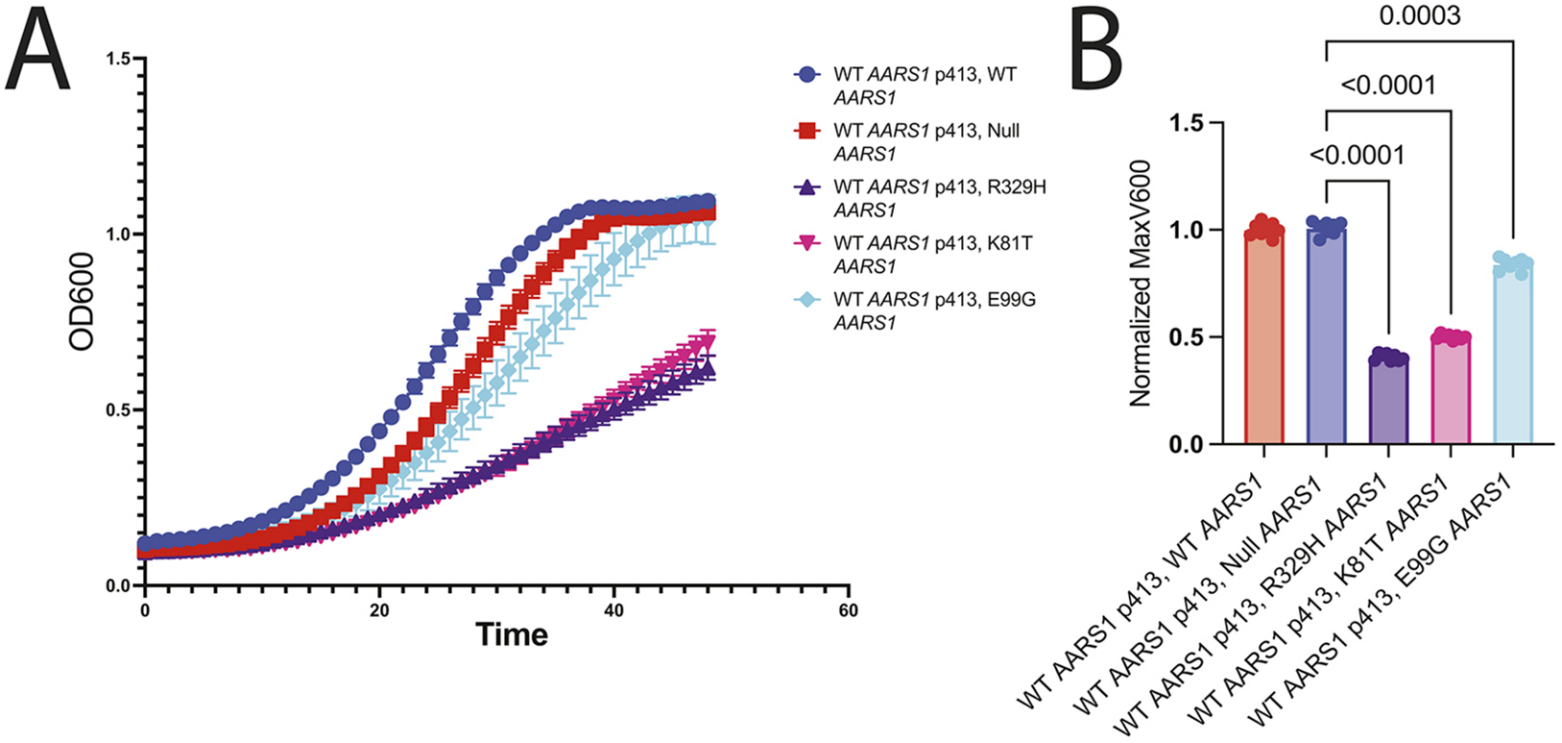
**Growth curve assessments of dominant-negative effects of *AARS1* missense variants in yeast.** Haploid yeast with a doxycycline-repressible endogenous *ALA1* (the yeast ortholog of *AARS1*) were transformed with p413 containing WT *AARS1*. Then, pAG425 vectors containing WT *AARS1* or mutant *AARS1*, or a pAG425 vector with a null allele (G757* *AARS1*) were transformed into the yeast strain. Cultures were diluted to a starting OD600 of 0.1 in 2% galactose/1% raffinose media lacking leucine and histidine with 100 µg/ml of doxycycline. Eight replicates were performed per experiment, and error bars represent s.d. (B) The MaxV600 was recorded from the growth curves, the background was subtracted, and readings were normalized to the average WT value in the presence of the *AARS1* null allele (G757*), which was set to one. Error bars represent s.d. Statistical significance was determined by one-way ANOVA with the Geisser-Greenhouse correction and Dunnett's multiple comparison test with individual variances computed for each comparison. Only comparisons that were statistically significant are annotated with a *P*-value.

K81T and E99G *AARS1* give rise to expressed, nonfunctional proteins. These observations raise the possibility that these two alleles have the potential to exert dominant-negative effects by interfering with the wild-type allele in the context of the AARS1 holoenzyme (a homodimer). We previously developed an assay to test for dominant-negative effects of *AARS1* variants by expressing mutant *AARS1* in the presence of wild-type *AARS1* and evaluating the effects on yeast cell growth (Meyer-Schuman et al., 2023). In brief, wild-type human *AARS1* on p413 and null (G757* *AARS1*), wild-type, or mutant human *AARS1* on pAG425 were transformed into the haploid ptetO7-*ALA1* yeast. Yeast growth was evaluated on medium containing doxycycline to repress endogenous *ALA1* expression and galactose to express the experimental allele; wild-type human *AARS1* was constitutively expressed on p413. When null *AARS1* on pAG425 was co-expressed with wild-type *AARS1* on p413, there was robust yeast cell growth, comparable to that when wild-type *AARS1* on pAG425 was co-expressed with wild-type *AARS1* on p413 (Fig. 5C,D; Fig. S8). This is consistent with wild-type human *AARS1* on p413 alone being sufficient to support yeast cell growth. When C901Y *AARS1* was co-expressed with wild-type *AARS1*, there was similar yeast cell growth to that observed when null *AARS1* was co-expressed with wild-type *AARS1* (Fig. 5C,D; Fig. S8), indicating that C901Y *AARS1* does not impact the function of wild-type *AARS1*; this is consistent with the reduced protein levels described above. In contrast, when K81T or E99G human *AARS1* were co-expressed with wild-type human *AARS1*, there was significantly reduced yeast cell growth compared to that with wild-type *AARS1* (Fig. 5C,D and Fig. 6; Figs S8 and S9). The reduction in yeast cell growth was reminiscent of that associated with R329H *AARS1* (Fig. 5C,D and Fig. 6; Figs S8 and S9), which causes dominant axonal neuropathy in several families (Meyer-Schuman et al., 2023). Importantly, the decreased yeast growth associated with co-expression of K81T or E99G human *AARS1* with wild-type human *AARS1* was rescued upon derepression of the endogenous *ALA1* yeast gene (Fig. S8B, middle column). This observation indicates that the reduced yeast growth is a direct effect of impaired alanyl-tRNA synthetase function and not due to off-target toxicity. In sum, our data suggest that K81T *AARS1* and E99G *AARS1* – variants implicated in recessive disease – have dominant-negative properties and that these alleles can cause axonal peripheral neuropathy in heterozygous carriers.

## DISCUSSION

In this study, we describe a humanized yeast assay to define and compare the functional consequences of 16 recessive-disease-associated cytoplasmic alanyl-tRNA synthetase (*AARS1*) variants. First, variants were tested in a yeast complementation assay using a high-copy number vector. Six variants (K81T, E99G, S188H, V666A, T726A and C901Y *AARS1*) displayed complete or partial loss-of-function effects. Next, the remaining variants that supported growth similarly to wild-type *AARS1* were tested in a yeast complementation assay using a low-copy number vector to detect more subtle effects on function. Four additional variants (G581S, N604K, I699T and G913D *AARS1*) displayed loss-of-function effects. Finally, variants that did not show a complete loss-of-function effect in either assay were tested in growth curve studies, which revealed one additional variant (L298Q *AARS1*) with a partial loss-of-function effect. Interestingly, there was no obvious correlation between conservation and functional effects in our yeast assay. For example, three of the variants that had no functional impact in any analysis (D530G, R751G and T756I) map to residues that are conserved in all seven species analyzed (Fig. 1B). Importantly, 11 out of 16 missense variants tested demonstrate loss-of-function effects in our yeast system, consistent with previous studies and supporting partial loss of function as the molecular mechanism of *AARS1*-associated recessive disease. Additionally, no patients carried two complete loss-of-function alleles, which is consistent with *AARS1* being essential for viability. Thus, yeast is an effective model for testing recessive *AARS1* alleles for complete or partial loss-of-function effects. Data from this model will be useful toward building arguments for the pathogenicity of newly discovered *AARS1* alleles, and similar studies should evaluate yeast as a model to study pathogenic alleles at other ARS loci.

That said, we noted two limitations in employing yeast to study *AARS1* alleles. First, this model was unable to detect loss-of-function effects for certain known pathogenic alleles. One genotype (Family B, R751G/R751G *AARS1*) consisted of homozygosity for one missense variant not demonstrating loss-of-function effects in our yeast assays; however, R751G is a highly confident pathogenic allele that has been identified in several patients and was predicted to be likely pathogenic by AlphaMissense (Table 2) (Cheng et al., 2023). Furthermore, *in vitro* aminoacylation assays previously showed that R751G *AARS1* results in a 10-fold decrease in tRNA charging (Table 2) (Simons et al., 2015), and aminoacylation activity in L298Q/R751G *AARS1* patient fibroblasts was 37% of that in controls (Marten et al., 2020). Thus, yeast models are not able to detect all loss-of-function effects of human pathogenic *AARS1* variants; this is likely due to the fact that yeast is a single-celled, rapidly dividing organism that may not reveal loss-of-function effects that occur in human tissues (Oprescu et al., 2017). To address these limitations, orthogonal approaches (e.g. enzyme kinetic assays) should be employed to assess for loss-of-function effects of *AARS1* alleles.

Second, our functional studies in yeast were unable to reveal relationships between *AARS1* gene dysfunction and clinical severity. The relationship between genotype and phenotype is challenging to evaluate given variability in clinical evaluations and difficulty in quantifying the severity of disease. Of the patients with bi-allelic missense variants, Family E (I699T/C901Y *AARS1*) had the most significant functional consequences in our assays, with C901Y *AARS1* demonstrating loss-of-function effects when modeled in the high-copy number vector and I699T *AARS1* showing reduced growth when modeled in the low-copy number vector; however, the patient phenotype was restricted to non-photosensitive trichothiodystrophy (Table 2) (Botta et al., 2021). Helman et al. (2021) described a series of patients (Families G-P in Table 2) with either early infantile-onset, severe (Families G-J) or later-onset, milder (Families K-P) recessive disease, and they found no correlation between the amount of decreased enzyme activity in fibroblast lysate aminoacylation studies and age of onset or severity. Our yeast studies also do not reveal a relationship between loss-of-function effects in yeast complementation assays and disease severity for these patients (Table 2). Two observations may explain the inability to accurately compare allelic differences among *AARS1* pathogenic variants, which might contribute to the lack of genotype–phenotype correlations. First, human *AARS1* does not completely rescue deletion of yeast *ALA1* (Fig. S1), which results in a limited functional range for comparing alleles. Second, the expression of yeast *ALA1* is not fully repressed in liquid media containing high levels of doxycycline, which limits the utility of quantitative growth curve analyses. Future studies could address these two issues through codon optimization and designing more effective strategies for repression of *ALA1*, respectively. In sum, although yeast is an effective model to study *AARS1* allele function, caution should be employed in interpreting negative data and in testing for genotype–phenotype correlations.

To identify potential dominant-negative *AARS1* alleles reported in patients with recessive disease, three variants (K81T, E99G and C901Y *AARS1*) that were unable to support any yeast cell growth were assessed for effects on protein expression and for the ability to impact the function of wild-type *AARS1*. These efforts revealed that two alleles (K81T and E99G) were expressed at levels comparable to those of the wild-type protein and resulted in reduced growth when co-expressed with wild-type *AARS1*, consistent with previously described dominant-negative *AARS1* alleles that cause axonal peripheral neuropathy (Meyer-Schuman et al., 2023). To our knowledge, no single *AARS1* variant has been implicated in both recessive and dominant diseases. Our data suggest that K81T and E99G *AARS1*, variants implicated in recessive disease, could also result in dominant axonal neuropathy in heterozygous carriers (i.e. parents and siblings of patients with the associated recessive phenotype). Furthermore, our results justify revisiting the clinical phenotype of the heterozygous parent in the affected pedigrees (Simons et al., 2015) and suggest that *AARS1*-associated phenotypes exist along a spectrum whereby peripheral neuropathy is caused by loss-of-function, dominant-negative alleles that decrease *AARS1* function with downstream effects on the integrated stress response (Spaulding et al., 2021), and that a multi-system syndrome is caused by two alleles with loss-of-function effects causing further reduction in *AARS1* function. In summary, this study has important implications for studying the allelic and clinical heterogeneity, and the molecular mechanisms, of *AARS1*-associated disease.

## MATERIALS AND METHODS

### Allele frequencies and conservation

The frequency of each variant was collected from gnomAD v4.0.0 (Lek et al., 2016; Karczewski et al., 2020). Conservation of each variant was examined by aligning AARS1 protein orthologs from multiple species with Clustal Omega. The accession numbers used were as follows: human (*Homo sapiens*), NP_001596.2; mouse (*Mus musculus*), NP_666329.2; zebrafish (*Danio rerio*), NP_001037775.1; fly (*Drosophila melanogaster*), AAF05593.1; worm (*Caenorhabditis elegans*), O01541.1; yeast (*Saccharomyces cerevisiae*), EDN63655.1; and bacteria (*Escherichia coli*), BAA16559.1.

### Yeast complementation assays

The 16 *AARS1* missense variants studied were modeled in the human *AARS1* open-reading frame. We performed site-directed mutagenesis using a sequence-verified, wild-type *AARS1* pDONR221 construct (Invitrogen), mutagenesis primers specifically designed for each *AARS1* missense variant (primer sequences available in Table S1) and the Quikchange II Site-Directed Mutagenesis Kit (Agilent). After transformation into bacteria, individual colonies were collected, and DNA was isolated. We next subjected each DNA sample to Sanger sequencing analysis to verify the *AARS1* variant sequence and the absence of unintended mutations that could arise from polymerase error. The sequence-verified expression constructs for wild-type and mutated *AARS1* were then cloned into either the pAG425 expression vector (pAG425GAL-ccdB; Addgene plasmid #14153; pAG425 harbors a galactose-inducible promoter) or the p413 expression vector (ATCC, 87370; p413 harbors the constitutive ADH promoter) using Gateway cloning technology (Invitrogen).

For expression of *AARS1* alleles from a high-copy number vector, null [G757* *AARS1*, for which no protein product is detectable by western blot analysis (Meyer-Schuman et al., 2023)], wild-type or mutant *AARS1* in pAG425 expression constructs were individually transformed into a haploid yeast strain, ptetO7-*ALA1* (p*ALA1*::kanR-tet07-TATA *URA3*::CMV-tTA *MATa*; from the Yeast Tet-Promoters Hughes Collection, Horizon Discovery, accession YSC1180), and subsequently plated on medium lacking leucine (pAG425 harbors the *LEU2* gene). Colonies were picked into liquid medium lacking leucine, grown at 30°C and shaken at 275 rpm for 48 h, then normalized to an OD600 of 2.0. Aliquots of these samples were diluted 1:10 and 1:100 on three plates (Takara Bio): (1) glucose plates lacking leucine (endogenous *ALA1* is expressed and *AARS1* on pAG425 is not expressed); (2) galactose/raffinose plates lacking leucine (endogenous *ALA1* and *AARS1* on pAG425 are both expressed); and (3) 2% galactose/1% raffinose plates lacking leucine and with 10 µg/ml doxycycline (endogenous *ALA1* is repressed and *AARS1* on pAG425 is expressed). Galactose concentrations of 0.2% and 0.02% were also tested, with wild-type *AARS1* rescuing becoming less noticeable (Figs S1 and S2).

For expression of *AARS1* alleles from a low-copy number vector, all experimental and control alleles (see above) in p413 constructs were individually transformed into the haploid yeast strain mentioned above and subsequently plated on medium lacking histidine (p413 harbors the *HIS3* gene). Colonies were picked into liquid medium lacking histidine, grown at 30°C and shaken at 275 rpm for 48 h, then normalized to an OD600 of 2.0. Aliquots of these samples were diluted 1:10 and 1:100 on two plates (Takara Bio): (1) glucose plates lacking histidine (endogenous *ALA1* is expressed and *AARS1* on p413 is expressed); and (2) glucose plates lacking histidine with 10 µg/ml doxycycline (endogenous *ALA1* is repressed and *AARS1* on p413 is expressed).

For dominant toxicity assays, null (G757* *AARS1*), wild-type or mutant *AARS1* in pAG425 expression constructs were transformed into the ptetO7-*ALA1* haploid yeast strain along with either empty or wild-type *AARS1* p413. Transformed yeast were plated on medium lacking leucine (pAG425 harbors the *LEU2* gene) and histidine (p413 harbors the *HIS3* gene). Colonies were picked into liquid medium lacking leucine and histidine, and normalized to an OD600 of 2.0. Aliquots were spotted on three plates: (1) glucose plates lacking leucine and histidine (endogenous *ALA1* is expressed, *AARS1* on p413 is expressed, and *AARS1* on pAG425 is not expressed); (2) galactose/raffinose plates lacking leucine and histidine (endogenous *ALA1*, *AARS1* on p413, and *AARS1* on pAG425 are all expressed); and (3) galactose/raffinose plates lacking leucine and histidine and with 10 µg/ml doxycycline (endogenous *ALA1* is repressed, *AARS1* on p413 is expressed, and *AARS1* on pAG425 is expressed).

After plating, yeast growth and viability were assessed visually after 5 days at 30°C. At least two independent transformations were performed, and at least three colonies per transformation were analyzed. Images of yeast spots were analyzed to assess the relative growth of mutant variants in comparison to wild-type *AARS1* using an established protocol (Petropavlovskiy et al., 2020). Images from the control (-his or -his-leu glucose) and experimental (-his or -his-leu galactose/raffinose with doxycycline) plates were imported to ImageJ. The image background was subtracted and assessed for uniformity across the image. The density of cells in each yeast spot (1:10 dilution) was individually measured. Growth for each spot on the experimental plate was calculated relative to the corresponding spot on the control plate. Relative growth for each mutant was then normalized to the growth of the wild-type spot on that plate. The average growth rate across three colony replicates for each mutant was calculated. Statistical significance was determined using GraphPad Prism, which performed one-way ANOVA with the Geisser-Greenhouse correction and Dunnett's multiple comparison's test with individual variances computed for each comparison.

### Yeast growth curve assay

For complementation assays, haploid yeast (ptet07-*ALA1*) were transformed with null (G757* *AARS1*), wild-type or mutant *AARS1* in pAG425 constructs as described above. Colonies were picked into 2 ml medium lacking leucine to select for the presence of pAG425 vector and grown at 30°C for 48 h, shaking at 275 rpm. The OD600 for saturated cultures was measured and diluted to a starting OD600 of 0.1 in liquid medium lacking leucine and containing 2% galactose/1% raffinose with 100 µg/ml doxycycline. Diluted cultures were aliquoted into a 96-well clear flat-bottom non-treated plate (Corning, 3370), covered with an optical overlay, and the OD600 was measured every hour for 72 h using a BioTek Epoch 2 Microplate Spectrophotometer. Doxycycline at 10 µg/ml and galactose at 0.2% and 0.02% were also tested (Fig. S3).

For dominant-negative assays, null (G757* *AARS1*), wild-type or mutant *AARS1* in pAG425 expression constructs were transformed into the ptetO7-*ALA1* haploid yeast strain along with either empty or wild-type *AARS1* p413. Colonies were picked into 2 ml medium lacking leucine and histidine to select for the presence of the pAG425 and p413 vectors, and grown at 30°C for 48 h, shaking at 275 rpm. The OD600 for saturated cultures was measured and diluted to a starting OD600 of 0.1 in liquid medium lacking leucine and containing 2% galactose/1% raffinose with 100 µg/ml doxycycline. Diluted cultures were aliquoted into a 96-well clear flat-bottom non-treated plate (Corning, 3370), covered with an optical overlay, and the OD600 was measured every hour for 48 h using a BioTek Epoch 2 Microplate Spectrophotometer. All yeast expression constructs are available upon request.

### Protein isolation and western blot analysis

Haploid yeast (ptetO7-*ALA1*) was transformed with wild-type, G757* or mutant *AARS1* pAG425 constructs as above. Colonies were picked into 5 ml galactose/raffinose liquid medium lacking leucine (endogenous *ALA1* and *AARS1* on pAG425 are expressed) and incubated at 30°C with shaking at 275 rpm for 2 days. Protein was isolated from yeast as previously described (Meyer-Schuman et al., 2023). Briefly, yeast cells were pelleted, supernatant was removed, and samples were frozen at −80°C. Samples were resuspended in 50 μl lysis buffer [50 mM Na-HEPES pH 7.5, 200 mM NaOAc, 1 mM EDTA, 0.25% NP-40, 3 mM DTT and 1× Halt Protease Inhibitor Cocktail (Thermo Fisher Scientific)]. Approximately 100 µl of 0.5 mm glass beads were added to each sample, and samples were vortexed at 4°C for 3 min, then incubated on ice for 3 min, then vortexed again for 3 min. The bottom of each 1.5 ml tube was punctured with a 26-guage needle, and the tube was placed into a 14 ml polypropylene round-bottom tube before centrifuging at 4°C for 5 min at 200 ***g***. The lysates were collected, and protein concentration was quantified using a Thermo Fisher Scientific Pierce BCA Protein Assay Kit.

For western blot analyses, 50 µg per sample was analyzed. Protein samples were prepared with 1× SDS sample buffer (Thermo Fisher Scientific) and 2-mercaptoethanol. Samples were denatured at 99°C for 5 min and separated on a 4-20% Tris-glycine protein gel (Thermo Fisher Scientific) at 150 V for 75 min. Protein was transferred to a methanol-treated polyvinylidene difluoride membrane (Millipore Sigma) using a Mini Trans Blot Electrophoretic Transfer Cell (Bio-Rad) at 100 V for 1 h. The membrane was blocked with 5% milk solution for 1 h at room temperature and then incubated overnight at 4°C in blocking solution containing primary antibodies, anti-AARS1 (Bethyl, A303-473A; 1:1000) and anti-PGK1 (Abcam, ab113689; 1:3000). The membrane was washed with 1× Tris-buffered saline solution and Tween 20 (TBST) for 5 min three times and then incubated with blocking solution containing IRDye 800CW goat anti-rabbit IgG secondary antibody (LI-COR), IRDye 680RD goat anti-mouse IgG secondary antibody (LI-COR), 0.02% SDS and 0.1% Tween 20 for 1 h at room temperature. The membrane was washed in 1× TBST for 5 min three times and imaged on a LI-COR Odyssey CLx Imager. Each experiment was performed three times. Band intensity was measured using ImageJ, and, for each sample, the intensity of the AARS1 band was calculated relative to the PGK1 band and then normalized to the intensity of the band for yeast transformed with the wild-type *AARS1* construct. Statistical significance was determined using GraphPad Prism, which performed one-way ANOVA with the Geisser-Greenhouse correction and Dunnett's multiple comparison's test with individual variances computed for each comparison.

## Supplementary Material

10.1242/dmm.052006_sup1 Supplementary information
