# Supplementary material for: Recessive, pathogenic AARS1 variants display variable loss-of-function and dominant-negative effects
Source: Dis Model Mech. 2025 Jun 27;18(6):dmm052006. doi: 10.1242/dmm.052006 (PMC12233060; doi:10.1242/dmm.052006)
Supplement: Supplementary information [file dmm-18-052006-s1.pdf]

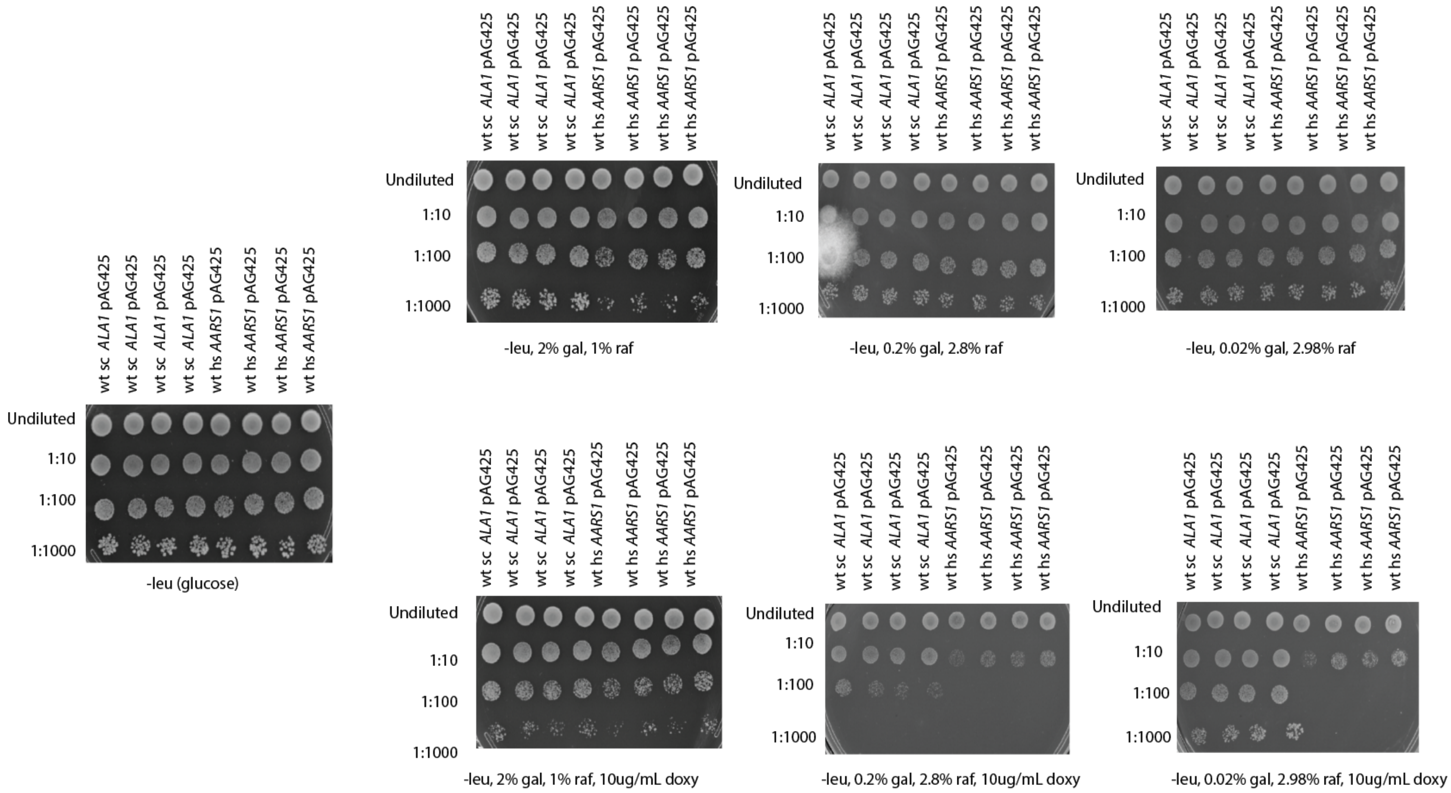

Fig. S1. Yeast complementation assay replicates and controls using a high copy number vector (pAG425) rescuing with human *AARS1* compared to rescuing with the yeast ortholog *ALA1*. Haploid yeast with a doxycycline-repressible endogenous *ALA1* (the yeast ortholog of *AARS1*) were transformed with pAG425 vectors containing wild-type (WT) yeast (sc) *ALA1* or wild-type human (hs) *AARS1*; the vector used in each experiment is indicated across the top. Resulting cultures were plated undiluted or diluted (1:10, 1:100, or 1:1000) on glucose media lacking leucine, different concentrations of galactose/raffinose media lacking leucine, or different concentrations of galactose/raffinose media lacking leucine and containing doxycycline. The different concentrations of galactose/raffinose are listed below each plate. Yeast was grown at 30°C for five days. Bacterial contamination was present on some plates but did not interfere with interpretation.

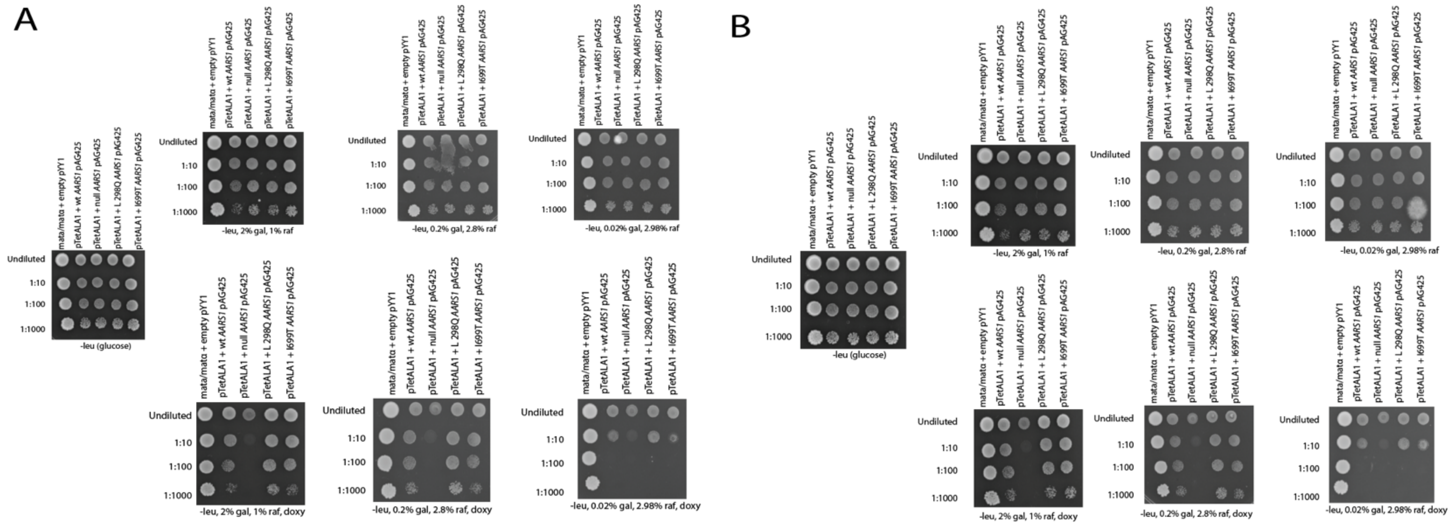

Fig. S2. Yeast complementation assay replicates using a high copy number, galactose-inducible vector (pAG425) on different concentrations of galactose. Diploid yeast that was transformed with an empty vector (pYY1) with a *LEU2* gene was used as a control to assess for yeast growth in relation to sugar content rather than absence or presence of the essential gene *ALA1*. Haploid yeast with a doxycycline-repressible endogenous *ALA1* (the yeast ortholog of *AARS1*) were transformed with pAG425 vectors containing wild-type (WT) *AARS1* or mutant *AARS1*, or a vector with a null allele (G757* *AARS1*); the yeast strain and vector used in each experiment is indicated across the top of each image. Resulting cultures were plated undiluted or diluted (1:10, 1:100, or 1:1000) on glucose media lacking leucine, different concentrations of galactose/raffinose media lacking leucine, or different concentrations of galactose/raffinose media lacking leucine and containing doxycycline. The different concentrations of galactose/raffinose are listed below each plate. Yeast was grown at 30°C for five days. Two independent replicates were generated (**A** and **B**). Bacterial contamination and smearing were present on some plates but did not interfere with interpretation.

Disease Models & Mechanisms • Supplementary information

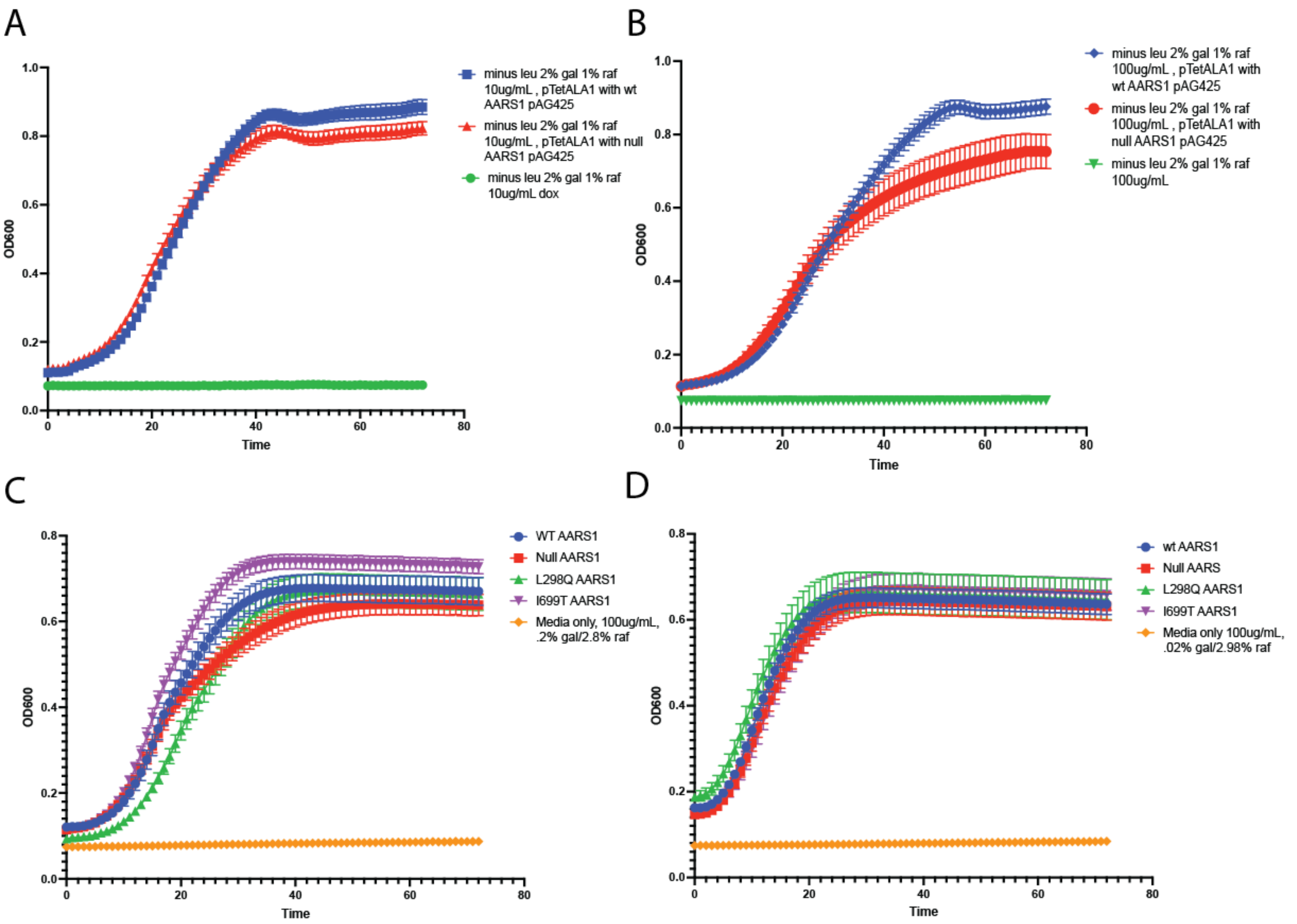

Fig. S3. Yeast growth curve replicates with controls using a high copy number, galactose inducible vector (pAG425). Haploid yeast with a doxycycline-repressible endogenous *ALA1* (the yeast ortholog of *AARS1*) were transformed with pAG425 vectors containing wild-type (WT) *AARS1* or mutant *AARS1*, or a pAG425 vector with a null allele (G757* *AARS1*). Resulting cultures were measured and then diluted to a starting OD600 of .1 in galactose/raffinose media lacking leucine with doxycycline. (**A**) Growth curve with 2% galactose/1% raffinose and 10ug/mL of doxycycline (**B**) Growth curve with 2% galactose/1% raffinose and 100ug/mL of doxycycline (**C**) Growth curve with .2% galactose/2.8% raffinose and 100ug/mL of doxycycline (**D**) Growth curve with .02% galactose/2.98% raffinose and 100ug/mL of doxycycline. Yeast were plated in a 96 well plate with each well receiving 150uL of diluted culture or media only. Growth was measured via OD600 every hour for 72 hours and then plotted with the mean of 8 replicates shown in the bold line and the standard deviation indicated by the bars. In each graph, wild-type *AARS1* is shown in blue, null (G757*) *AARS1* is shown in red, media is shown in yellow, and the variants are shown in various colors.

Disease Models & Mechanisms • Supplementary information

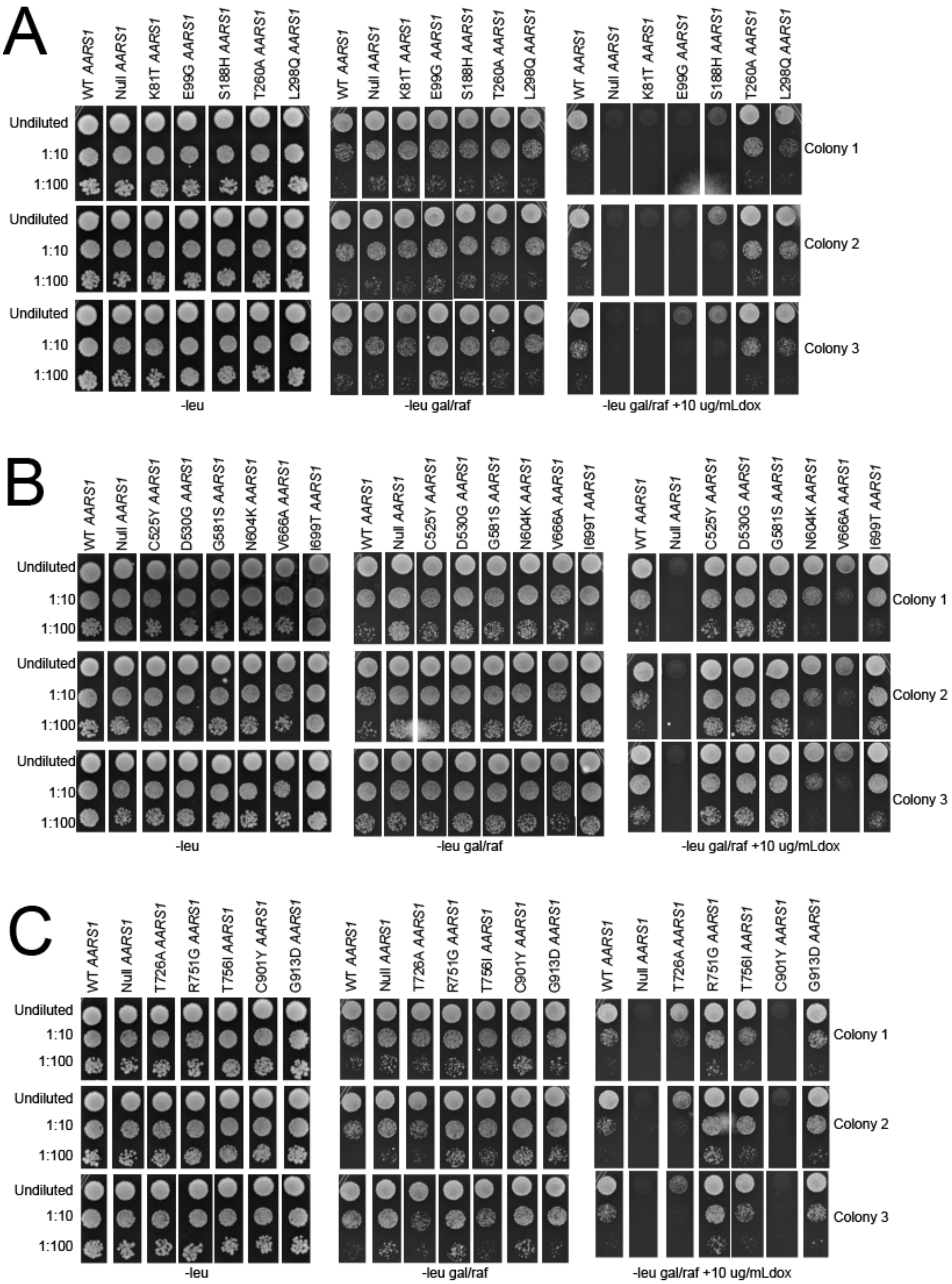

Fig. S4. Yeast complementation assay replicates and controls using a high copy number vector (pAG425). Haploid yeast with a doxycycline-repressible endogenous *ALA1* (the yeast ortholog of *AARS1*) were transformed with pAG425 vectors containing wild-type (WT) *AARS1* or mutant *AARS1*, or a vector with a null allele (G757* *AARS1*); the vector used in each experiment is indicated across the top. Resulting cultures were plated undiluted or diluted (1:10 or 1:100) on glucose media lacking leucine, 2% galactose/1% raffinose media lacking leucine, or 2% galactose/1% raffinose media lacking leucine and containing 10ug/mL doxycycline. Yeast was grown at 30°C for five days. Variants were divided into three sets (**A**, **B**, **C**). Bacterial contamination was present on some plates but did not interfere with interpretation.

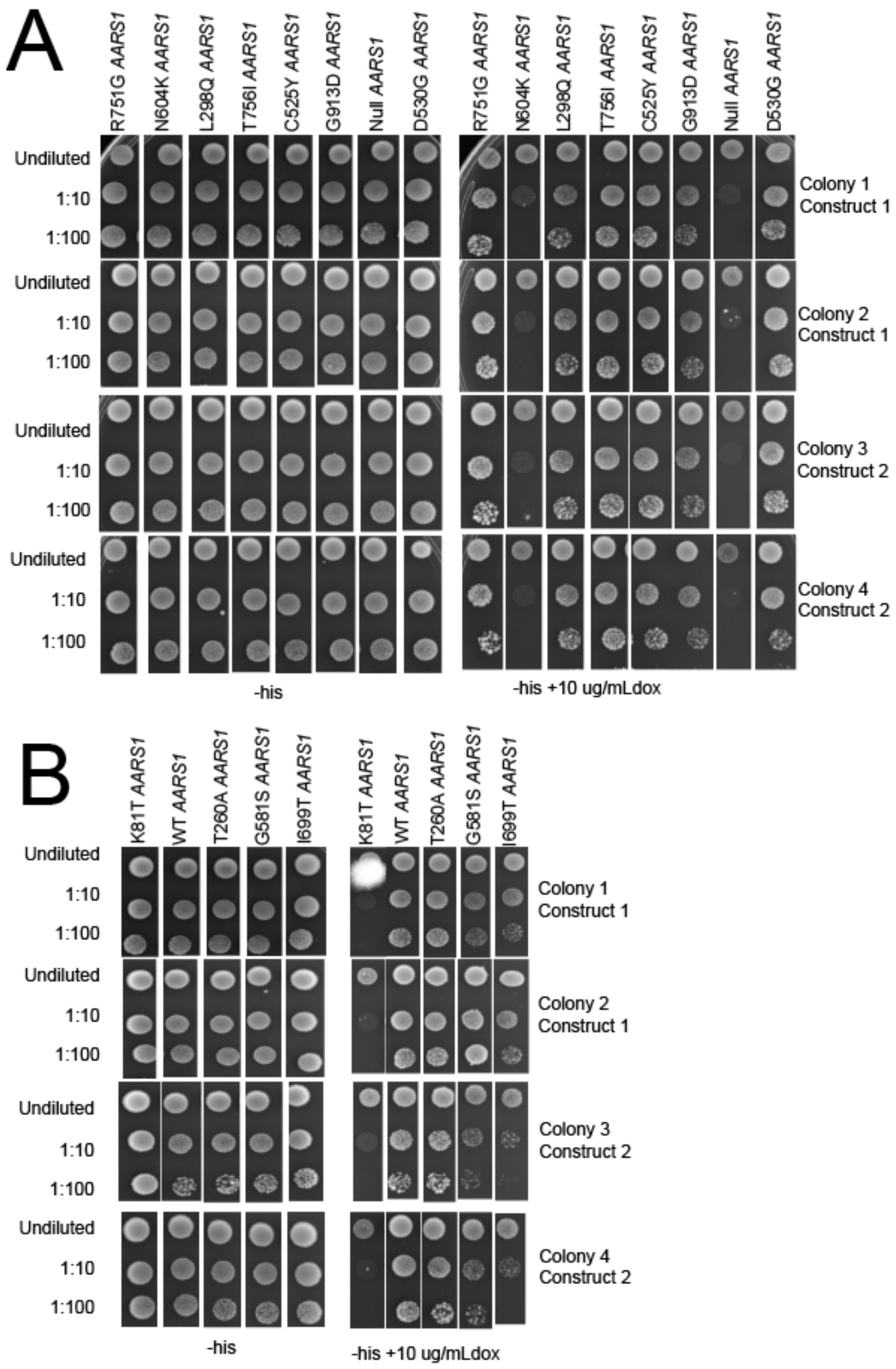

Fig. S5. Yeast complementation assay replicates and controls using a low copy number vector (p413). Haploid yeast with a doxycycline-repressible endogenous *ALA1* (the yeast ortholog of *AARS1*) were transformed with p413 vectors containing wild-type (WT) *AARS1* or mutant *AARS1*, or a p413 vector with a null allele (G757* *AARS1*); the vector used in each experiment is indicated across the top. Resulting cultures were plated undiluted or diluted (1:10 or 1:100) on glucose media lacking histidine or glucose media lacking histidine and containing 10 ug/mL doxycycline. Yeast was grown at 30°C for five days. Variants were spotting across two plates (**A**, **B**). Bacterial contamination was present on some plates but did not interfere with interpretation.

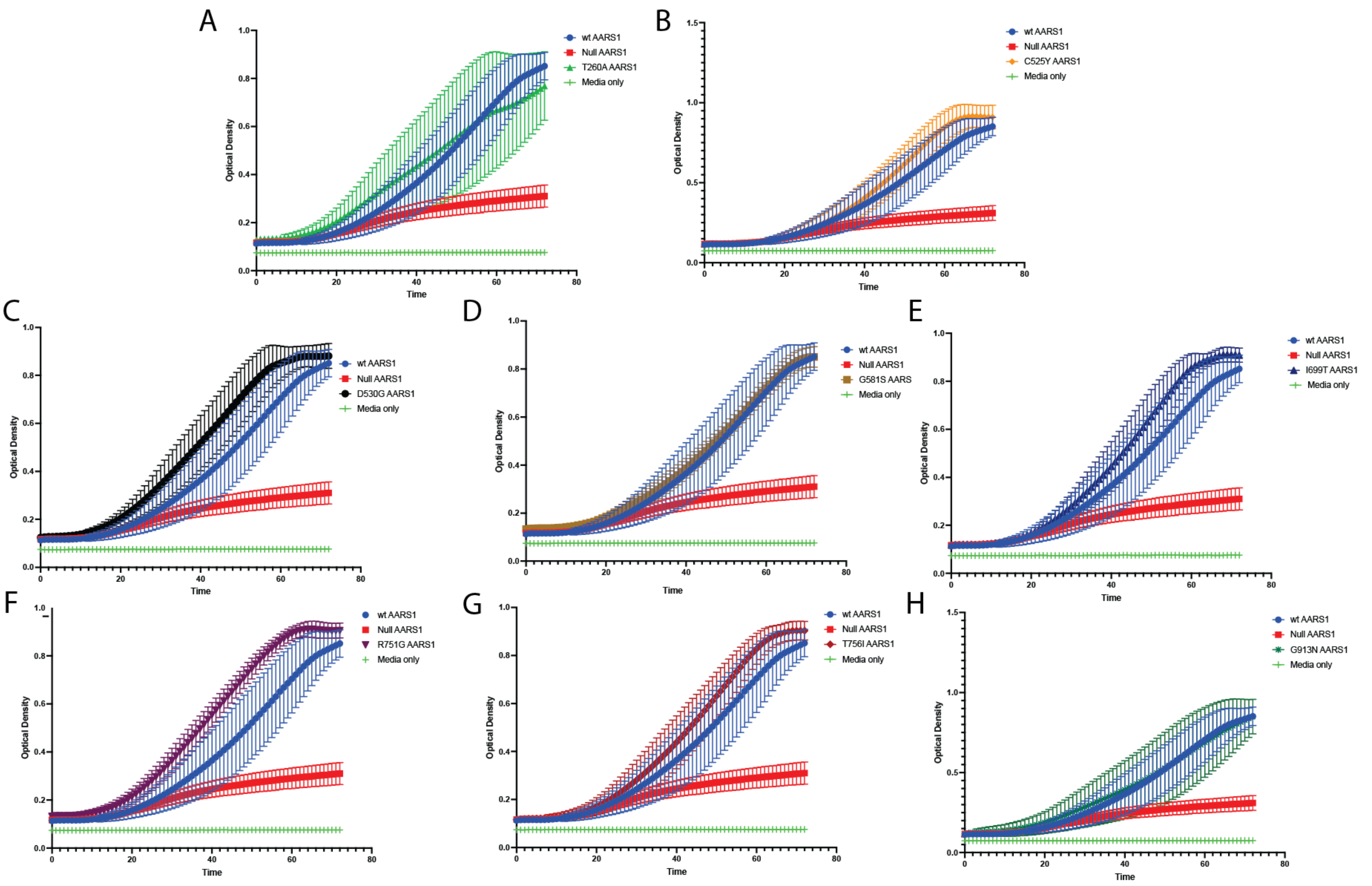

Fig. S6. Yeast growth curve replicates with controls using a high copy number, galactose inducible vector (pAG425). Haploid yeast with a doxycycline-repressible endogenous *ALA1* (the yeast ortholog of *AARS1*) were transformed with pAG425 vectors containing wild-type (WT) *AARS1* or mutant *AARS1*, or a pAG425 vector with a null allele (G757* *AARS1*). Resulting cultures were measured and then diluted to a starting OD600 of .1 in 2% galactose/1% raffinose media lacking leucine with 100ug/mL of doxycycline. Yeast were plated in a 96 well plate with each well receiving 150uL of diluted culture or media only where each plate had 8 replicates per variant per plate with each variant being tested in two plates. Growth was measured via OD600 every hour for 72 hours and then plotted with the mean of 16 replicates shown in the bold line and the standard deviation indicated by the bars. In each graph, wild-type *AARS1* is shown in blue, null (G757*) *AARS1* is shown in red, media is shown in green, and the variants are shown in various colors.

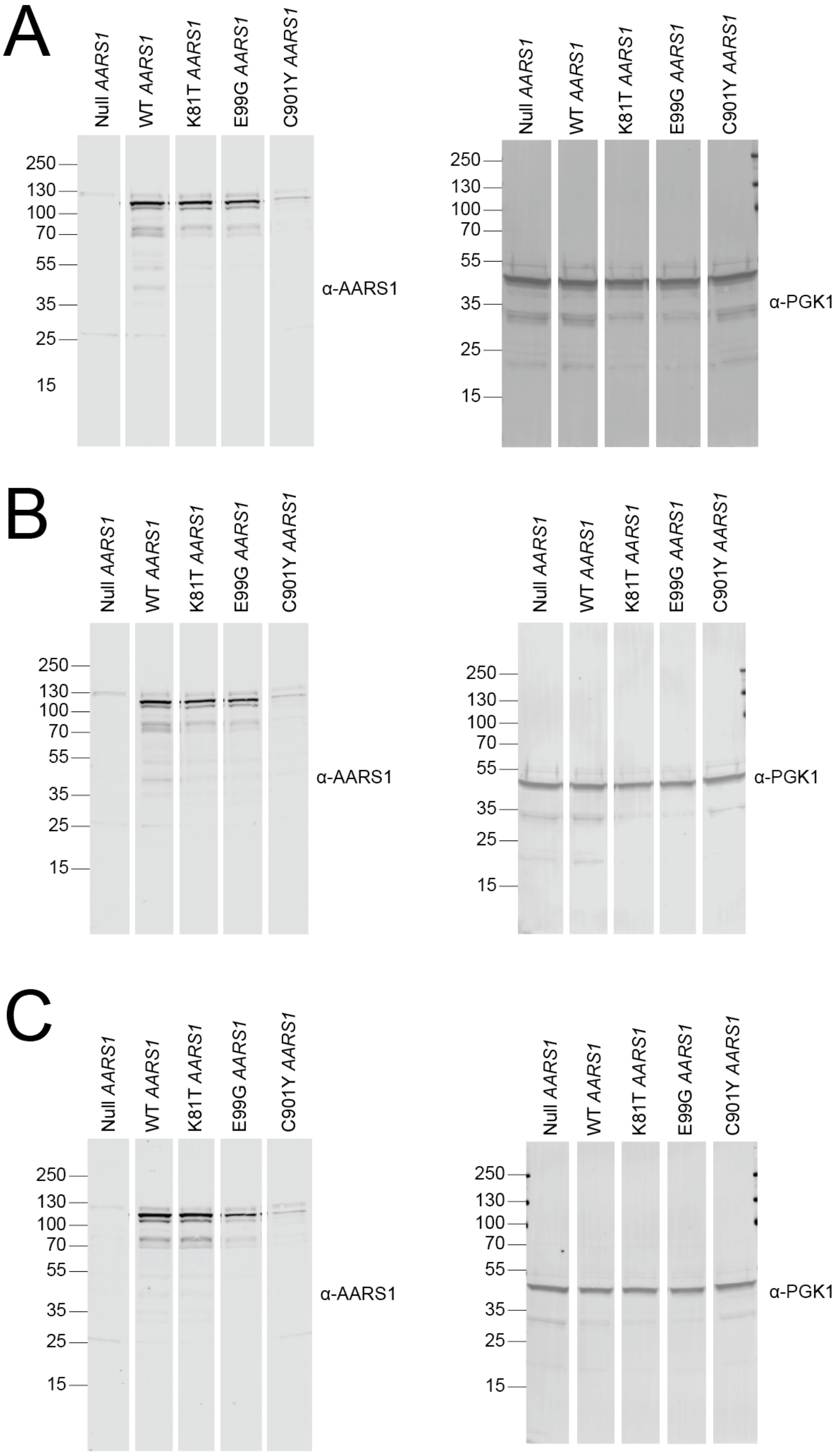

Fig. S7. Replicates and full blots of western blot experiments. Western blot analyses were performed using protein lysates isolated from haploid yeast that were transformed with the pAG425 vector indicated across the top and antibodies to AARS1 and PGK1.

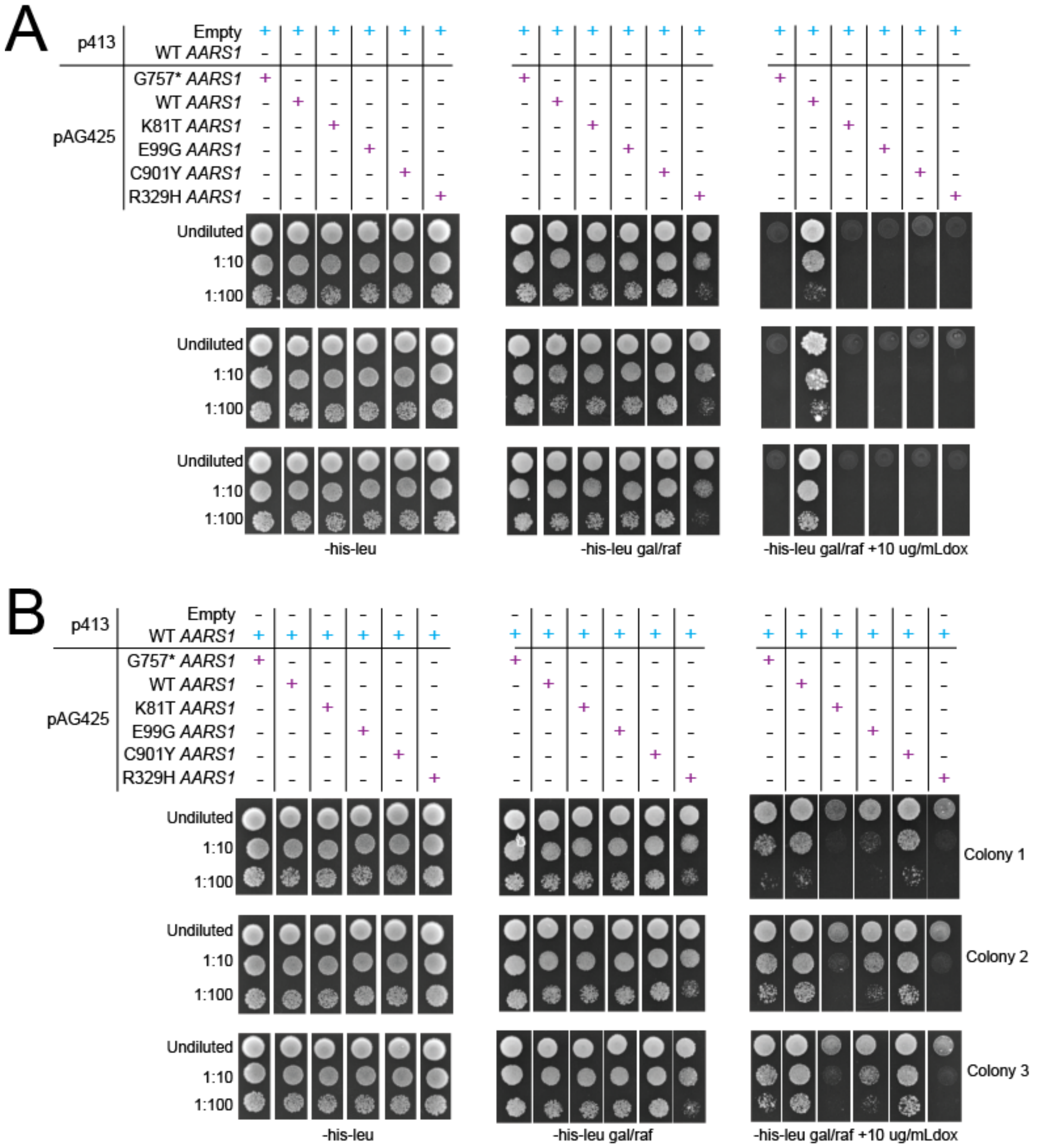

Fig. S8. Replicates and control plates for yeast complementation assays co-expressing empty or wild-type *AARS1* p413 constructs with mutant *AARS1* pAG425 constructs. Haploid yeast with a doxycycline-repressible endogenous *ALA1* (the yeast ortholog of *AARS1*) containing a wild-type (WT) *AARS1* or empty p413 construct were transformed with pAG425 vectors containing the indicated insert. Resulting cultures were plated undiluted or diluted (1:10 or 1:100) on glucose media lacking histidine and leucine, galactose/raffinose media lacking histidine and leucine, or galactose/raffinose media lacking histidine and leucine and containing doxycycline. Yeast were grown at 30°C for five days.

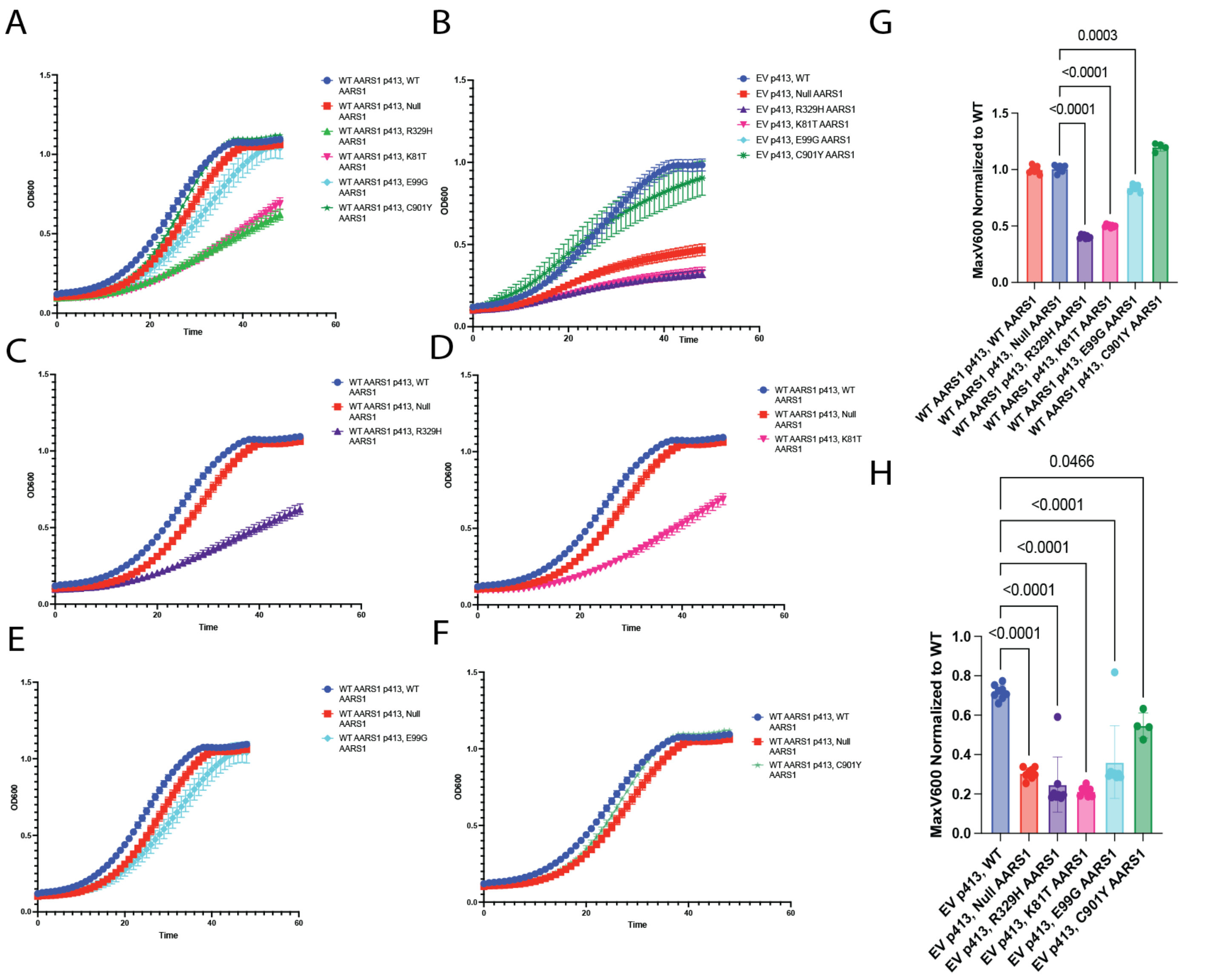

Fig. S9. Yeast growth curves for dominant toxicity assay. Haploid yeast with a doxycycline-repressible endogenous *ALA1* (the yeast ortholog of *AARS1*) were transformed with p413 vectors that were empty (EV) or contain wild-type *AARS1* (WT). Then, pAG425 vectors containing wild-type (WT) *AARS1* or mutant *AARS1*, or a pAG425 vector with a null allele (G757* *AARS1*) were transformed into the yeast in addition to the p413 vector. Yeast was grown in media lacking histidine and leucine to select for the vectors. Resulting cultures were measured and then diluted to a starting OD600 of 0.1 in 2% galactose/1% raffinose media lacking leucine and histidine with 100ug/mL of doxycycline. Yeast were plated in a 96 well plate with each well receiving 150uL of diluted culture or media only where each plate had 8 replicates per variant with the exception of C901Y *AARS1* which had 4 replicates. (a-f) Growth was measured via OD600 every hour for 72 hours and then plotted with the mean of 16 replicates shown in the bold line and the standard deviation indicated by the bars. In each graph, wild-type *AARS1* is shown in blue, null (G757*) *AARS1* is shown in red, media is shown in green, and the variants are shown in various colors. (g and h) The max OD600 (MaxV600) were recorded from the growth curves, the background was subtracted and then normalized to the average wild-type in the presence of the null or wild-type in the presence of EV on the plate which was set to one. Error bars represent standard deviation. Statistical significance was determined by one-way ANOVA with the Geisser-Greenhouse correction and Dunnett's multiple comparison's test with individual variances computed for each comparison. Only comparisons that were statistically significant comparisons are annotated with the p-value.

Disease Models & Mechanisms • Supplementary information

Table S1. *AARS1* mutagenesis primers.

Variant	Forward Primer	Reverse Primer
K81T	TCCGGGCTGGGGGCACACATAATGACCTGGA	TCCAGGTCATTATGTGTGCCCCCAGCCCGGA
E99G	ATCACACCTTCTTCGGGATGCTGGGCTCTTG	CAAGAGCCCAGCATCCCGAAGAAGGTGTGAT
S188H	CCCTGTGGTCCTTGCCATGAGATCCACTACGA	TCGTAGTGGATCTCATGGCAAGGACCACAGGG
T260A	ATGTCCAACTATGACGCTGACCTTTTTGTCC	GGACAAAAAGGTCAGCGTCATAGTTGGACAT
L298Q	TGGCCTACCGGGTGCAGGCTGACCACGCTCG	CGAGCGTGGTCAGCCTGCACCCGGTAGGCCA
C525Y	CCACAGGCCAGGAGTATGGAGTGGTGCTGGA	TCCAGCACCACTCCATACTCCTGGCCTGTGG
D530G	GTGGAGTGGTGCTGGGCAAGACCTGTTTCTA	TAGAAACAGGTCTTGCCCAGCACCACTCCAC
G581S	ATTGGAACCATCTACAGTGACCTGAAAGTGG	CCACTTTCAGGTCACTGTAGATGGTTCCAAT
N604K	ACCCATCATGAGCAAGCACACAGCTACGCAC	GTGCGTAGCTGTGTGCTTGCTCATGATGGGT
V666A	AGGCAGCCAAGGCCGCCTATACCCAGGATTG	CAATCCTGGGTATAGGCGGCCTTGGCTGCCT
I699T	TGCGAGTCGTCTCCACTGGGGTCCCGGTGTC	GACACCGGGACCCCAGTGGAGACGACTCGCA
T726A	GAGTTCTGTGGGGGAGCGCACCTGCGGAACT	AGTTCCGCAGGTGCGCTCCCCCACAGAACTC
R751G	GCCAAGGGTATCCGGGGGATTGTGGCTGTCA	TGACAGCCACAATCCCCCGGATACCCTTGGC
T756I	GGATTGTGGCTGTCATAGGTGCCGAGGCCCA	TGGGCCTCGGCACCTATGACAGCCACAATCC
C901Y	CTGGCAAGATCACGTACCTGTGTCAAGTTCC	GGAACTTGACACAGGTACGTGATCTTGCCA
G913D	ATGCAGCCAATCGGGACTTAAAAGCCAGCGA	TCGCTGGCTTTTAAGTCCCGATTGGCTGCAT

Disease Models & Mechanisms • Supplementary information
